# ATGL is a biosynthetic enzyme for fatty acid esters of hydroxy fatty acids

**DOI:** 10.1038/s41586-022-04787-x

**Published:** 2022-06-08

**Authors:** Rucha Patel, Anna Santoro, Peter Hofer, Dan Tan, Monika Oberer, Andrew T. Nelson, Srihari Konduri, Dionicio Siegel, Rudolf Zechner, Alan Saghatelian, Barbara B. Kahn

**Affiliations:** 1 Division of Endocrinology, Diabetes and Metabolism, Department of Medicine, Beth Israel Deaconess Medical Center and Harvard Medical School, Boston, MA USA; 2 Institute of Molecular Biosciences, University of Graz, Graz, Austria; 3 Clayton Foundation Laboratories for Peptide Biology, Salk Institute for Biological Studies, La Jolla, CA USA; 4 Skaggs School of Pharmacy and Pharmaceutical Sciences, University of California-San Diego, La Jolla, CA USA; 5 BioTechMed-Graz, Graz, Austria

**Keywords:** Fatty acids, Fat metabolism

## Abstract

Branched fatty acid (FA) esters of hydroxy FAs (HFAs; FAHFAs) are recently discovered lipids that are conserved from yeast to mammals^1,2^. A subfamily, palmitic acid esters of hydroxy stearic acids (PAHSAs), are anti-inflammatory and anti-diabetic^1,3^. Humans and mice with insulin resistance have lower PAHSA levels in subcutaneous adipose tissue and serum^1^. PAHSA administration improves glucose tolerance and insulin sensitivity and reduces inflammation in obesity, diabetes and immune-mediated diseases^1,4–7^. The enzyme(s) responsible for FAHFA biosynthesis in vivo remains unknown. Here we identified adipose triglyceride lipase (ATGL, also known as patatin-like phospholipase domain containing 2 (PNPLA2)) as a candidate biosynthetic enzyme for FAHFAs using chemical biology and proteomics. We discovered that recombinant ATGL uses a transacylation reaction that esterifies an HFA with a FA from triglyceride (TG) or diglyceride to produce FAHFAs. Overexpression of wild-type, but not catalytically dead, ATGL increases FAHFA biosynthesis. Chemical inhibition of ATGL or genetic deletion of *Atgl* inhibits FAHFA biosynthesis and reduces the levels of FAHFA and FAHFA-TG. Levels of endogenous and nascent FAHFAs and FAHFA-TGs are 80–90 per cent lower in adipose tissue of mice in which *Atgl* is knocked out specifically in the adipose tissue. Increasing TG levels by upregulating diacylglycerol acyltransferase (DGAT) activity promotes FAHFA biosynthesis, and decreasing DGAT activity inhibits it, reinforcing TGs as FAHFA precursors. ATGL biosynthetic transacylase activity is present in human adipose tissue underscoring its potential clinical relevance. In summary, we discovered the first, to our knowledge, biosynthetic enzyme that catalyses the formation of the FAHFA ester bond in mammals. Whereas ATGL lipase activity is well known, our data establish a paradigm shift demonstrating that ATGL transacylase activity is biologically important.

## Abstract (web summary)

A study in mammals identifies a new role for adipose triglyceride lipase in catalysing the esterification of hydroxyl fatty acids to produce biologically active fatty acid esters of hydroxy fatty acids.

## Main

Branched FAHFAs are present in all mammalian tissues studied with the highest levels in white adipose tissue (WAT) and brown adipose tissue (BAT)^1^. Forty-six FAHFA families have been identified in mouse WAT^8^ and even more in human WAT^3^, which have a unique composition of FAs and HFAs. In human WAT, these consist of 583 regioisomers^3^ that are defined by the position of the ester bond between the HFA and the FA. FAHFAs are highly regulated in a tissue-specific manner in physiological and pathophysiological states. Subcutaneous (SQ) WAT FAHFA levels increase rapidly with fasting^1^. PAHSA levels in SQ WAT and serum correlate positively with insulin sensitivity in humans and mice, and levels are reduced before overt diabetes occurs in people with insulin resistance^1^. Specific PAHSA isomers are reduced in the breast milk of mothers who are obese^9^, and serum levels are decreased in patients with breast cancer^10^. Exercise training induces serum and WAT PAHSA levels in elderly women^11^. Levels of circulating palmitoleic acid ester of 9-HSA (9-POHSA) and oleic acid ester of 9-HSA (9-OAHSA) correlate with protective cardiovascular biomarkers in healthy humans^12^. Thus, FAHFAs have the potential to be important in many physiological and pathophysiological states in humans.

The biological effects of only a few FAHFAs are known. Administration of 5- and 9-PAHSA improves glucose tolerance and insulin sensitivity in mice by multiple mechanisms including enhancing insulin-mediated suppression of FA release from WAT, and improving insulin action to suppress hepatic glucose production^7^. PAHSA treatment also decreases WAT inflammation^1^ and improves cognitive function in obese mice^13^. In addition, 5- and 9-PAHSA treatment reduces the incidence of auto-immune type 1 diabetes^6^ and decreases the severity of colitis^4^ in mice. PAHSAs and polyunsaturated FAHFA family members have anti-inflammatory effects directly on immune cells^1,14,15^. 9-Palmitic acid hydroxy palmitic acid and 9-oleic acid hydroxy palmitic acids seem to increase basal metabolism and insulin sensitivity in mice^16,17^. Thus, FAHFAs are endogenous lipids with broadly beneficial metabolic and anti-inflammatory properties.

Little is known about the molecular mechanisms regulating FAHFAs. Whereas four hydrolytic enzymes have been identified—carboxyl ester lipase^18^, androgen‐dependent tissue factor pathway inhibitor regulating protein, androgen-induced gene 1 (ref. ^19^) and hormone-sensitive lipase^20^—no enzymes that catalyse the formation of the ester bond between the FA and the HFA are known in an intact organism. Substrate availability seems to regulate FAHFA levels in vivo. For example, increased WAT glucose uptake and de novo lipogenesis markedly increases FAHFA levels in WAT and serum in mice overexpressing GLUT4 specifically in adipose tissue (AG4OX mice)^1^. Administration of C17-HFA to mice promotes FAHFA biosynthesis^1^, and availability of HFAs synthesized by cellular anti-oxidation pathways regulates FAHFA levels, as WAT PAHSA levels are reduced in peroxiredoxin 6 (*Prdx6*)-knockout (KO) mice^21^. Furthermore, supplementation of humans and mice with marine oils containing omega-3 polyunsaturated FAs increases FAHFAs consisting of these lipids, again demonstrating substrate regulation of FAHFA levels^14^. Other organisms can also synthesize FAHFAs from exogenous FAs. This is demonstrated by the fact that communal yeast strains that lack the FA synthesis gene, *Fasn*, and are dependent on exogenous FAs from human skin for survival, have endogenous FAHFAs^2^. In this study, we aim to discover the enzyme(s) responsible for FAHFA biosynthesis (that is, the formation of the ester bond between the FA and the HFA). Understanding how FAHFAs are synthesized could lead to therapies to maintain or restore FAHFA levels in people with metabolic or immune-mediated disorders.

## Higher FAHFA synthesis in AG4OX adipocytes

**Fig. 1:**
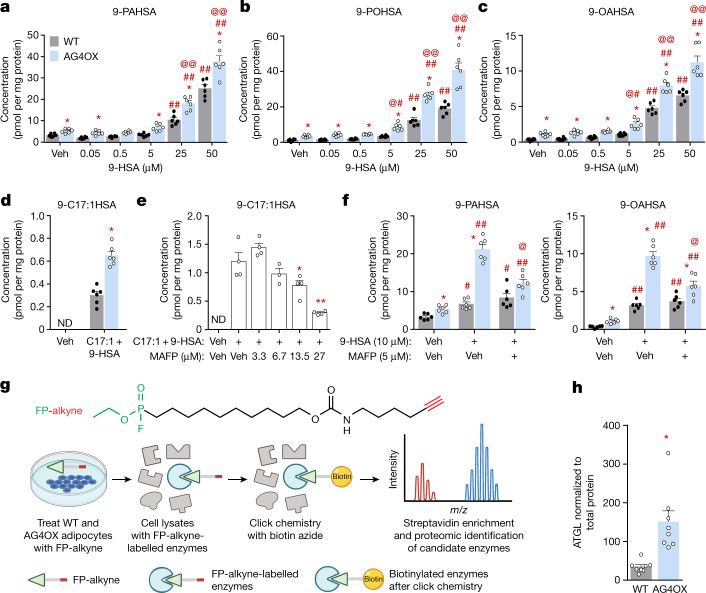
FAHFA biosynthesis is increased in AG4OX SVF adipocytes and is sensitive to fluorophosphonate inhibitors. **a**–**d**, Biosynthesis of 9-PAHSA (**a**), 9-POHSA (**b**), 9-OAHSA (**c**) and 9-C17:1HSA (**d**) in WT and AG4OX SVF adipocytes incubated with 0.1% DMSO (vehicle (Veh)), increasing concentrations of 9-HSA (**a**–**c**) or both C17:1 and 9-HSA (25 µM, each) (**d**). *n* = 6 wells per condition with cells pooled from 9 mice per genotype; mean ± s.e.m. *P < 0.003 versus WT, same treatment (*t*-test correcting for Holm–Sidak multiple comparisons). #*P* < 0.05, ###*P* < 0.0001 versus vehicle, same genotype, @*P* < 0.05, @@*P* < 0.0001 versus WT, same treatment (two-way analysis of variance (ANOVA)). ND, not detected. **e**, 9-C17:1HSA biosynthesis in 3T3-L1 adipocytes pre-incubated with the indicated concentrations of MAFP or 0.1% DMSO (Veh) for 1 h, and then co-incubated with C17:1 and 9-HSA (10 µM each) or 0.1% DMSO (Veh) for 2 h. *n* = 4 wells except for MAFP 6.75 µM group *n* = 3; mean ± s.e.m. *P < 0.05, **P < 0.0001 versus C17:1 and 9-HSA alone (one-way ANOVA). **f**, Biosynthesis of 9-PAHSA (left) and 9-OAHSA (right) in WT and AG4OX SVF adipocytes pre-incubated with MAFP or 0.1% DMSO for 1 h, and then co-incubated with 9-HSA (10 µM) or 0.1% DMSO (Veh) for 2 h. *n* = 6 wells per condition; mean ± s.e.m. *P < 0.003 versus WT, same treatment (*t*-test correcting for Holm–Sidak multiple comparisons). #*P* < 0.05, ##*P* < 0.0001 versus Veh, @*P* < 0.0001 versus 9-HSA alone, same genotype (two-way ANOVA). **g**, Structure of FP-alkyne (top) and schematic diagram of activity-based proteomics (bottom). Created with BioRender.com. **h**, ATGL protein levels in AG4OX SQ WAT, normalized to total protein between 37–25 kDa on western blot. *n* = 8 mice, mean ± s.e.m. *P < 0.0012 versus WT (*t*-test, two-tailed). Similar results were obtained in at least two independent experiments. See also Extended Data Fig. 1.

FAHFA levels are 8–16-fold higher in WAT of AG4OX mice^1^. We reasoned that this may result from upregulation of FAHFA biosynthesis. Basal levels of 9-PAHSA, 9-POHSA and 9-OAHSA are 1.5–3.4-fold higher in adipocytes derived from the stromal vascular fraction (SVF) of WAT of AG4OX mice (Fig. 1a–c). 9-hydroxy stearic acid (9-HSA) addition increased 9-FAHFA biosynthesis in a dose-dependent manner in both wild-type (WT) and AG4OX adipocytes (Fig. 1a–c). FAHFA biosynthesis was 35–50% higher in AG4OX compared to WT adipocytes at all HSA concentrations. *Cis*-10-heptadecenoic (C17:1) FA is a low-abundance endogenous FA that allows us to measure FAHFA production without a large background signal. It also ensures that differences in the biosynthetic activity are not due to different concentrations of FAs between AG4OX and WT cells. Addition of 9-HSA and C17:1 resulted in a twofold increase in 9-C17:1-HSA in AG4OX compared to WT cells (Fig. 1d). Thus, adipocytes from AG4OX mice have increased FAHFA biosynthetic activity, providing a cellular model to identify the specific biosynthetic enzymes and pathways.

## Fluorophosphonates inhibit FAHFA synthesis

**Extended Data Fig. 1:**
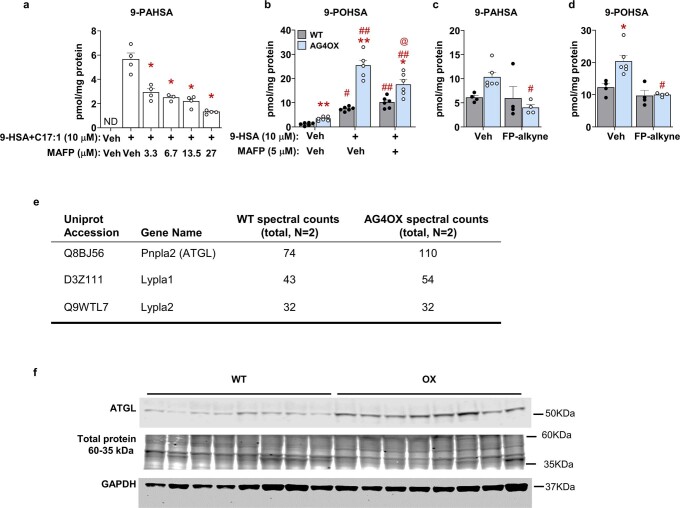
(related to Figure 1): (a-e) FAHFA biosynthesis is sensitive to fluorophosphonate inhibitors; (f) ATGL protein is increased in AG4OX mice adipose tissue. **(a)** 9-PAHSA biosynthesis in 3T3-L1 adipocytes pre-incubated with the indicated concentrations of methyl arachidonyl fluorophosphonate (MAFP) or 0.1% DMSO (Veh) for 1hr, and then co-incubated with C17:1 and 9-HSA or 0.1% DMSO (Veh) for 2hrs. (n = 4 wells, except for MAFP 6.75 µM group n = 3, mean±SEM), *P < 0.0001 vs. C17:1 and 9-HSA only group (One-way ANOVA) **(b)** 9-POHSA biosynthesis in WT and AG4OX SVF-adipocytes pre-incubated with MAFP or 0.1% DMSO for 1hr, then co-incubated with 9-HSA or 0.1% DMSO (Veh) for 2hrs. (n = 6 wells, mean±SEM) *P < 0.05, **P < 0.0001 vs. WT, same treatment, t-test correcting for Holm-Sidak multiple comparisons. #P < 0.001, ##P < 0.0001 vs. the Veh for 9-HSA, same genotype, @P < 0.05 vs. 9-HSA alone, same genotype (Two-way ANOVA). **(c)** 9-PAHSA and **(d)** 9-POHSA biosynthesis in WT and AG4OX SVF-adipocytes pre-incubated with FP-alkyne (5 µM) or 0.1% DMSO for 1hr, then co-incubated with 9-HSA (10 µM) for 2hrs. (n = 4 except for AG4OX Vehicle group = 6, mean±SEM) *P < 0.001 vs. WT, same treatment, #P < 0.01 vs. Veh, same genotype (Two-way ANOVA). **(e)** Selected list of candidate FAHFA biosynthetic enzymes from activity-based proteomics study in WT and AG4OX SVF adipocytes. **(f)** WT and AG4OX SQ WAT western blots for ATGL and GAPDH. Total protein between 37 and 25 kDa was visualized by Revert stain (N = 8/genotype). Loading control, GAPDH, and ATGL were run on the same blot. This blot is representative of 3 separate blots. ND means not detected.

Methyl arachidonyl fluorophosphonate (MAFP) is an irreversible, covalent inhibitor of serine^22,23^ and threonine^24^ lipases. Fluorophosphonate inhibitors, similar to MAFP, inhibit all four lipases that hydrolyse FAHFAs in vitro and in vivo^19,25^. We first tested MAFP as a FAHFA hydrolysis inhibitor in 3T3-L1 adipocytes incubated with C17:1 FA and 9-HSA to measure biosynthesis without competing effects from hydrolysis. Blocking FAHFA hydrolysis should increase FAHFA levels. Instead, we observed a dose-dependent decrease in 9-C17:1HSA (Fig. 1e) and 9-PAHSA (Extended Data Fig. 1a) levels with MAFP addition. Increased biosynthesis of 9-PAHSA, 9-OAHSA (Fig. 1f) and 9-POHSA (Extended Data Fig. 1b) in AG4OX adipocytes is also reduced (50%) by MAFP. Owing to MAFP specificity, the data indicate that an enzyme(s) with an active-site serine nucleophile mediates FAHFA biosynthesis. Several lipid-synthesizing enzyme classes have serine nucleophiles including acyltransferases and transacylases^22^.

To identify the enzyme(s) responsible for increased 9-FAHFA biosynthesis in AG4OX adipocytes, we performed activity-based protein profiling using a fluorophosphonate (FP)-alkyne probe^23,25^ that uses the same functional group as MAFP and therefore should inhibit the same enzymes. Covalent labelling with the probe labels the enzyme with an alkyne that can be used to enrich and detect the target proteins through proteomics (Fig. 1g). Like MAFP, FP-alkyne blocked the increase in 9-PAHSA and 9-POHSA biosynthesis in AG4OX, but not WT, adipocytes (Extended Data Fig. 1c, d), validating that one or more FP-alkyne targets is involved in FAHFA biosynthesis.

We performed click chemistry with biotin azide to biotinylate the FP-alkyne target proteins in lysates from FP-alkyne-treated WT and AG4OX adipocytes and pulled down these proteins with streptavidin agarose^25^. The resulting samples were analysed by proteomics. We performed gene ontology analysis to filter the candidate proteins for enzymes involved in lipid metabolism, and then further truncated this list to include only proteins with an active-site serine nucleophile. These are listed in Extended Data Fig. 1e. ATGL had a robust proteomics signal and was increased by 1.5-fold in the AG4OX samples compared to WT, which we validated by western blotting (Fig. 1h and Extended Data Fig. 1f). ATGL, which catalyses the first step of TG hydrolysis to release FAs, is a critical regulator of lipid metabolism^26^.

## Inhibiting ATGL decreases FAHFA synthesis

**Fig. 2:**
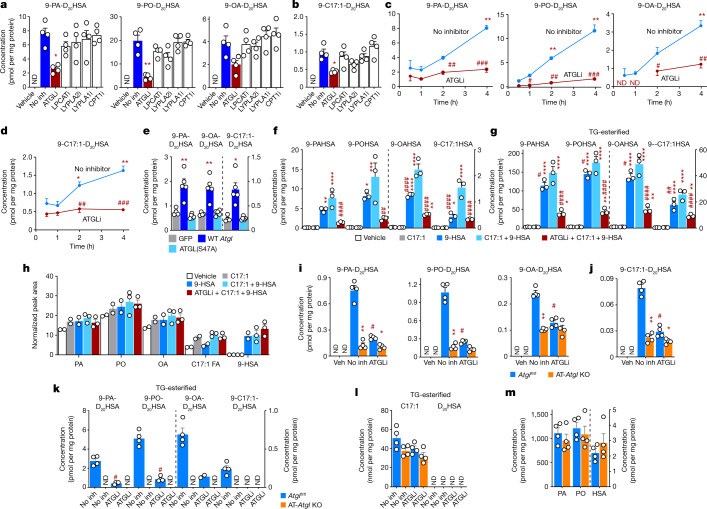
ATGL regulates biosynthesis of FAHFA and TG-esterified FAHFA in intact cells. Cells were incubated with both C17:1 FA and D_20_-9-HSA (20 µM (**a**–**d**, **i**–**l**) or 25 µM (**e**–**h**) each) or 0.05% DMSO (vehicle) for 4 h. **a**, **b**, Biosynthesis of 9-PA-D_20_HSA, 9-PO-D_20_HSA and 9-OA-D_20_HSA (**a**) and 9-C17:1-D_20_HSA (**b**) was measured in WT SVF adipocytes pre-incubated with the indicated enzyme inhibitors or 0.1% DMSO (No inh). LPCATi, lysophosphatidylcholine acyltransferase inhibitor; LYPLA2i, lysophospholipase 2 inhibitor; LYPLA1i, lysophospholipase 1 inhibitor; CPT1i, carnitine palmitoyltransferase 1 inhibitor. *n* = 4 wells; mean ± s.e.m. *P <  0.001, **P < 0.0001 versus No inh group (one-way ANOVA). **c**, **d**, Biosynthesis of 9-PA-D_20_HSA, 9-PO-D_20_HSA and 9-OA-D_20_HSA (**c**) and 9-C17:1-D_20_HSA (**d**) in WT SVF adipocytes co-incubated with atglistatin (ATGL inhibitor (ATGLi), 10 µM) or 0.1% DMSO (No inh) for 0.5–4 h. *n* = 4; mean ± s.e.m. *P < 0.05, **P < 0.0001 versus 0.5 h, same treatment, #*P* < 0.05, ##*P* < 0.001, ###*P* < 0.0001 versus No inh, same time point (two-way ANOVA). **e**, Biosynthesis of 9-PA-D_20_HSA, 9-OA-D_20_HSA and 9-C17:1-D_20_HSA in HEK293T cells transfected with GFP, WT ATGL and ATGL(S47A) mutant enzymes. *n* = 4, mean ± s.e.m. *P < 0.01, **P < 0.001 versus GFP-transfected cells (one-way ANOVA). **f**–**h**, Biosynthesis of 9-PAHSA, 9-POHSA, 9-OAHSA and 9-C17:1HSA (**f**), biosynthesis of TG-esterified 9-FAHFAs (**g**) and levels of FFA and 9-HSA (**h**) in 3T3-L1 adipocytes incubated with C17:1 or 9-HSA alone or with or without atglistatin (10 µM). *n* = 3; mean ± s.e.m. *P < 0.05, **P < 0.01, ***P < 0.001, ****P < 0.0001 versus vehicle, #*P* < 0.05, ##*P* < 0.01, ####*P* < 0.0001 versus C17:1 + 9-HSA group (one-way ANOVA). **i**–**m**, Biosynthesis of 9-PA-D_20_HSA, 9-PO-D_20_HSA and 9-OA-D_20_HSA (**i**), biosynthesis of 9-C17:1-D_20_HSA (**j**), biosynthesis of TG-esterified 9-FA-D_20_HSA (**k**), levels of TG-esterified C17:1 and D_20_HSA (**l**) and levels of FFA and HSA (**m**) in AT-*Atgl*-KO and *Atgl*^*fl/fl*^ SVF adipocytes incubated with atglistatin (10 µM) or 0.1% DMSO (No inh). TG-esterified 9-FA-D_20_HSA levels were measured in *n* = 4 samples per condition. FFA and HSA levels were measured in the Veh condition; *n* = 4 mean ± s.e.m. *P < 0.05, **P < 0.0001 versus WT, same treatment, #*P* < 0.0001 versus No inh, same genotype (two-way ANOVA, **i**, **j**, *t*-test two-tailed, **k**). Levels above the limit of quantification are shown with white circles. Similar results were obtained in two independent experiments.

MAFP-sensitive enzymes involved in lipid metabolism from our proteomics experiment were tested in WT adipocytes using the following enzyme-specific inhibitors: atglistatin (ATGL inhibitor), TSI-01 (lysophosphatidylcholine acyltransferase inhibitor), ML-348 (lysophospholipase 1 inhibitor), ML-349 (lysophospholipase 2 inhibitor) and etomoxir (carnitine palmitoyltransferase 1 inhibitor). After adipocytes were incubated with D_20_-9-HSA and C17:1 FA, the ATGL inhibitor atglistatin reduced incorporation of D_20_-9-HSA into different FAHFAs consisting of endogenous FAs or C17:1 FA by 50–80% (Fig. 2a, b). All other inhibitors tested were inactive. A time-course experiment showed a cumulative increase in newly synthesized FAHFAs up to 4 h and this is reduced in atglistatin-treated cells as early as 1 h, supporting the role of ATGL in FAHFA biosynthesis (Fig. 2c, d).

## ATGL overexpression increases FAHFA synthesis

**Extended Data Fig. 2:**
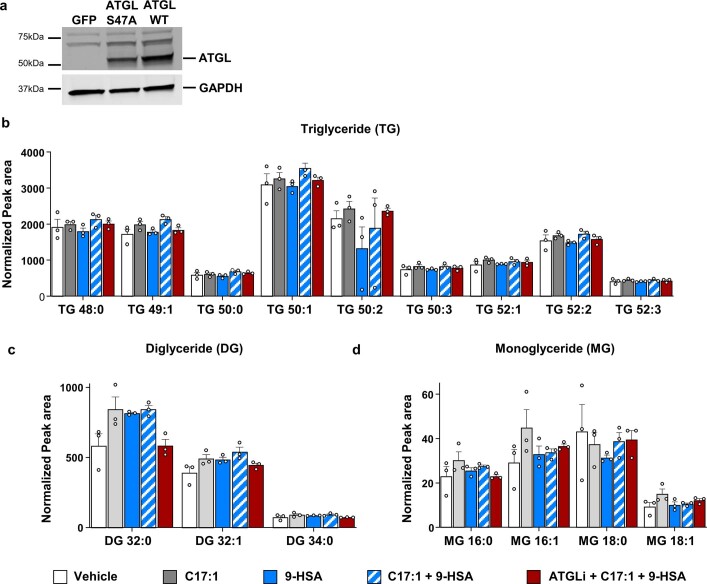
(related Figure 2): Effect of ATGL inhibition on FAHFA biosynthesis and cellular glycerolipids. **(a)** Western-blots confirming overexpression of catalytically dead ATGL S47A and ATGL WT in HEK293T cells. Similar results were obtained in 3 independent experiments **(b)** Triglyceride **(c)**, diglyceride and **(d)** monoglyceride levels in 3T3-L1 adipocytes incubated with C17:1 (25 µM), 9-HSA (25 µM) with or without atglistatin (ATGLi, 10 µM) or 0.1%DMSO. n = 3 wells, mean ± SEM.

To further demonstrate the importance of ATGL as a mediator of FAHFA biosynthesis, we overexpressed WT ATGL or the catalytically inactive serine mutant ATGL(S47A) in HEK293T cells and measured FAHFA biosynthesis. Both constructs were efficiently overexpressed (Extended Data Fig. 2a). Overexpression of WT ATGL increased FAHFA biosynthesis activity whereas enzymatically dead ATGL(S47A) did not (Fig. 2e). Thus, ATGL activity is sufficient to regulate FAHFA biosynthesis.

## ATGL inhibition decreases FAHFA-TGs not glycerolipids

FAHFAs are incorporated into triglycerides (FAHFA-TGs)^27^. ATGL mediates FAHFA release from FAHFA-TGs during lipolysis^20,27,28^. To investigate whether loss of ATGL activity lowers FAHFA levels by trapping FAHFAs in FAHFA-TGs or alternatively by modulating substrate concentrations, we measured the levels of FAs, 9-HSA, FAHFAs, FAHFA-TGs and glycerolipids in the biosynthesis experiment. Atglistatin inhibited FAHFA biosynthesis by 76–81% (Fig. 2f). This was not due to accumulation of FAHFA-TGs, which were also diminished 67–78% (Fig. 2g). Thus, ATGL inhibition results in reduction of FAHFAs and FAHFA-TGs. Free FA (FFA) (Fig. 2h), triglyceride, diglyceride and monoglyceride levels (Extended Data Fig. 2b–d) were not changed in the presence of atglistatin, indicating that the marked reduction in FAHFA biosynthesis with ATGL inhibition is not a result of substrate depletion. These data demonstrate that ATGL regulates FAHFA biosynthesis through a biochemical mechanism that differs from the lipolytic effects of ATGL.

## *Atgl* KO decreases FAHFA and FAHFA-TG synthesis

**Extended Data Fig. 3:**
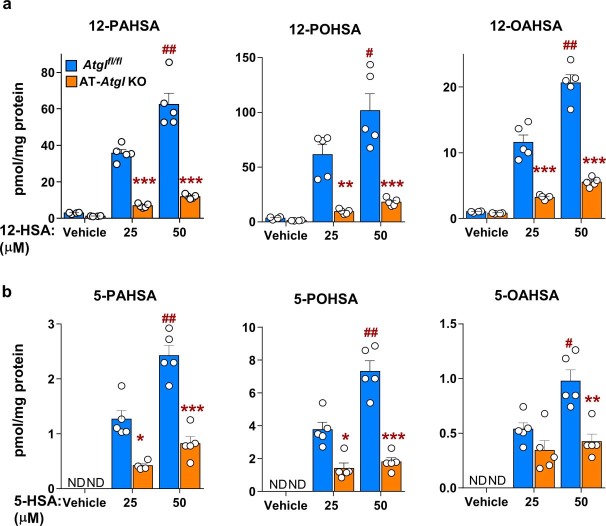
ATGL regulates FAHFA biosynthesis in intact cells. **(a)** 12-PAHSA, 12-POHSA, 12-OAHSA, **(b)** 5-PAHSA, 5-POHSA and 5-OAHSA biosynthesis in *Atgl*^fl/fl ^and AT-*Atgl*-KO SVF-derived adipocytes incubated with 12-HSA, 5-HSA or 0.05% DMSO (Vehicle) for 4hrs. n = 5 wells, mean±SEM. *P < 0.002, **P  = 0.001, ***P < 0.0001, vs. *Atgl*^fl/fl^, same treatment, #P < 0.01, ##P < 0.0001 vs. 25µM HSA same genotype (Two-way ANOVA **a-b**). ND means not detected.

Biosynthesis of 9-FAHFAs (Fig. 2i, j), 12-FAHFAs (Extended Data Fig. 3a) and 5-FAHFAs (Extended Data Fig. 3b) is 60–84% reduced in adipocytes in which *Atgl* has been knocked out in the adipose tissue (AT-*Atgl*-KO adipocytes) versus *Atgl*^*fl/fl*^ adipocytes (Fig. 2i, j), consistent with the atglistatin results. Nascent FAHFA-TGs are not detected in AT-*Atgl*-KO cells (Fig. 2k), suggesting that non-esterified FAHFAs are synthesized before they are esterified into TGs. Atglistatin has no impact on FAHFA biosynthesis in AT-*Atgl*-KO adipocytes (Fig. 2i–k), confirming atglistatin specificity for ATGL inhibition. During the study C17:1 FA is incorporated into triglyceride as an acyl chain, but not D_20_-9-HSA (Fig. 2l). We cannot rule out that D_20_-9-HSA or 9-HSA might be incorporated into TGs under different conditions. *Atgl*^*fl/fl*^ and AT-*Atgl*-KO adipocytes have similar levels of endogenous FFAs (PA, PO and HSA) (Fig. 2m). These data indicate that ATGL activity is necessary for FAHFA biosynthesis in adipocytes and loss of activity by small-molecule inhibition or genetic deletion reduces FAHFA biosynthesis. Furthermore, the reduction in FAHFA biosynthesis with ATGL inhibition or *Atgl* KO does not result from insufficient FFAs levels.

## ATGL transacylation increases FAHFA synthesis

Having ruled out the possibility that FAHFAs are getting trapped in TGs, the data suggest that ATGL synthesizes FAHFAs through esterification of HSAs to FAs. In addition to its TG hydrolase activity^29–31^, ATGL has retinyl ester hydrolase activity^32^ and phospholipase activity^33^, and produces triglycerides from monoglycerides and diglycerides through a CoA-independent transacylase reaction^30,34^ in vitro. An ATGL transacylase activity could provide a mechanism for FAHFA biosynthesis, but the aforementioned reaction was observed only in vitro and has not been tested for FAHFA biosynthesis. ATGL is a member of the patatin-like phospholipase domain containing (PNPLA) family^26^. A family member, PNPLA1 has physiologically important transacylase activity that transfers an acyl chain from TG to a hydroxy-ceramide in the skin^35,36^.

**Fig. 3:**
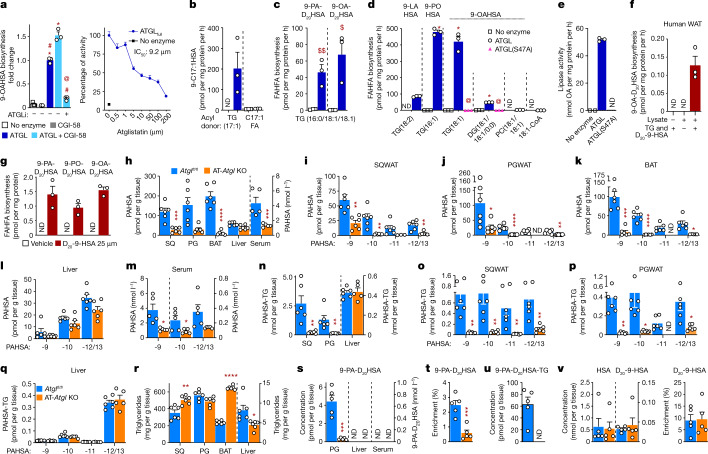
ATGL transacylation activity synthesizes FAHFAs in vitro and in vivo. **a**, Left, biosynthesis of 9-OAHSA in vitro by affinity-purified WT ATGL-288 (ATGL) and the cofactor CGI-58 from 9-HSA and acyl-donor TG(18:1) and inhibition with atglistatin (50 µM). Right, inhibition of the transacylation activity of affinity-purified full-length ATGL (ATGL_full_) by the indicated doses of atglistatin. **b**, **c**, WT ATGL-288 catalysed biosynthesis of 9-C17:1HSA from 9-HSA and acyl donors TG(C17:1) and C17:1(FFA) (**b**) and biosynthesis of 9-PA-D_20_HSA and 9-OA-D_20_HSA from D_20_-9-HSA and acyl donor TG(C16:0/18:1/18:1) (**c**). **d**, WT ATGL-288 and ATGL-288(S47A) transacylation activity catalysed 9-FAHFA biosynthesis from acyl donors: TG(18:2), TG(16:1), TG(18:1), DG(C18:1/18:1/0:0), phosphatidylcholine (PC(18:1/18:1/0:0)) and 18:1-CoA. **e**, Lipase activity of WT ATGL-288 (ATGL) and ATGL-288(S47A) enzymes as measured by OA release during the FAHFA biosynthesis assay with the substrate TG(18:1) in **d**. **f**, 9-OAHSA biosynthesis from TG(18:1) and D_20_-9-HSA in human SQ WAT lysates. **g**, 9-FAHFA biosynthesis in human SVF adipocytes incubated with D_20_-9-HSA or vehicle (0.025% DMSO). (*n* = 3, mean ± s.e.m. **a**–**g**), *P < 0.0001 versus no enzyme, #*P* < 0.0001 versus ATGL/CGI-58 and @*P* < 0.0001 versus ATGL (one-way ANOVA, **a**, **d**). $*P* <  0.02, $$*P* < 0.008 versus no enzyme (*t*-test, two-tailed **c**). AT-*Atgl*-KO and littermate *Atgl*^*fl/fl*^ female mice were used for **h**–**v**. **h**–**k**, Total endogenous PAHSA (**h**) and PAHSA isomer levels in SQ WAT (**i**), PG WAT (**j**), BAT (**k**), liver (**l**) and serum (**m**). **n**–**q**, Total TG-esterified PAHSA (**n**) and TG-esterified PAHSA isomer levels in SQ WAT (**o**), PG WAT (**p**) and liver (**q**). **r**, Adipose tissue and liver total triglyceride levels. AT-*Atgl*-KO and *Atgl*^*fl/fl*^ mice were injected with D_20_-9-HSA (5 mg kg^−1^) intraperitoneally and euthanized 4 h later (**s**–**v**). **s**, PG WAT, liver and serum levels of newly synthesized 9-PA-D_20_HSA. **t**–**v**, 9-PA-D_20_HSA enrichment (9-PA-D_20_HSA per cent of total 9-PAHSA) (**t**), levels of newly synthesized TG-esterified 9-PA-D_20_HSA (**u**), endogenous HSA substrate level (**v**, left) and enrichment of D_20_-9-HSA (**v**, right) in PG WAT. *n* = 6 mice for all tissues in **h**–**r**; *n* = 5 mice for serum in **h**, **m** and **s**–**v**; mean ± s.e.m. *P < 0.05, **P < 0.01, ***P < 0.001, ****P < 0.0001 versus *Atgl*^*fl/fl*^ mice *t*-test, two-tailed (**h**–**v**). White error bars are shown within the bars in **o**, **p**. Only levels above the quantification limit are shown with white circles. Source data

**Extended Data Fig. 4:**
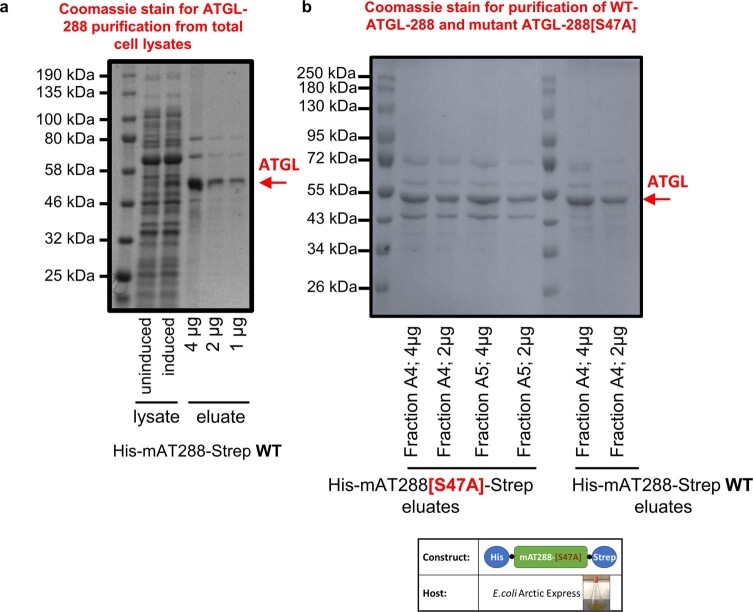
(related to Figure 3 a-e) Coomassie stain of affinity-purified ATGL-288 WT and ATGL-288[S47A] mutant enzymes. **(a)** Coomassie-stained SDS gel showing induction and purification of ATGL-288 WT enzyme from *E. coli* Arctic Express. **(b)** Coomassie-stained SDS gel showing affinity-purified WT ATGL-288 and mutant ATGL-288[S47A] enzymes. Depiction of ATGL-288[S47A] plasmid construct and host e-coli culture (bottom panel).

To test whether ATGL can esterify HSAs, we incubated an affinity-purified truncation variant of ATGL including amino acids 1–288 (ATGL-288) (Extended Data Fig. 4a, b) and comparative gene identification-58 (CGI-58), an ATGL cofactor that increases its basal activity^37^, with 9-HSA and a triglyceride acyl donor, TG(18:1). ATGL-288 is a recently described form of ATGL that retains biological activity which is augmented by CGI-58 and inhibited by ATGLi^38^. We used ATGL-288 for all but one experiment because we can obtain large quantities of this owing to optimization of the purification and expression of this form of ATGL in bacteria^38^. CGI-58 had no FAHFA biosynthetic activity by itself (Fig. 3a, left). ATGL increased 9-OAHSA biosynthesis by 13-fold using TG as an acyl donor, and addition of CGI-58 further increased this transacylation activity by another 53% (Fig. 3a, left). Atglistatin inhibited this biosynthetic activity by 85%. This was a dose-dependent effect, underscoring the specificity of the activity for ATGL (Fig. 3a, right). The half-maximal inhibitory concentration (IC_50_) of atglistatin for FAHFA biosynthesis catalysed by the full-length ATGL enzyme is 9.2 µM (Fig. 3a, right). ATGL catalysed robust 9-C17:1HSA biosynthesis in the presence of TG(17:1) but not C17:1 FFA (Fig. 3b). This indicates that ATGL transacylation activity is required for FAHFA biosynthesis and FAs are not direct precursors of FAHFA biosynthesis. ATGL synthesized both 9-PA-D_20_HSA and 9-OA-D_20_HSA in the presence of acyl donor TG(16:0/18:1/18:1) with D_20_-9-HSA (Fig. 3c). ATGL also catalysed 9-FAHFA biosynthesis from acyl donors TG(16:1) and TG(18:1) and, to a lesser extent, from TG(18:2) and diglyceride(18:1/18:1/0:0) (Fig. 3d). The catalytically dead mutant ATGL(S47A) did not produce FAHFAs in vitro (Fig. 3d), consistent with the lack of FAHFA biosynthesis catalysed by this mutant ATGL in cells (Fig. 2e). Acyl donors phosphatidylcholine(18:1/18:1) and OA-CoA are unable to provide acyl chains for ATGL to catalyse FAHFA biosynthesis (Fig. 3d). ATGL preference for TG acyl chains 16:0, 16:1 and 18:1 as substrates for transacylation-catalysed FAHFA biosynthesis is similar (Fig. 3c, d). The turnover rate of free FAHFA is about 420 pmol per mg protein per hour in our biosynthesis assay with ATGL-288 enzyme with acyl donor TG(18:1) (Fig. 3d). The turnover rate of free OA is about 52 nmol mg^−1^ protein per hour in our assay conditions (Fig. 3e).

These results show that the transacylase activity of ATGL synthesizes FAHFAs from di- and tri-glyceride acyl donors and HFAs. Furthermore, the same FAHFA biosynthesis pathway is active in human adipose tissue lysates (Fig. 3f), and human adipocytes (Fig. 3g) can synthesize FAHFAs from triglycerides and D_20_-9-HSA.

## ATGL regulates FAHFA levels in vivo

**Extended Data Fig. 5:**
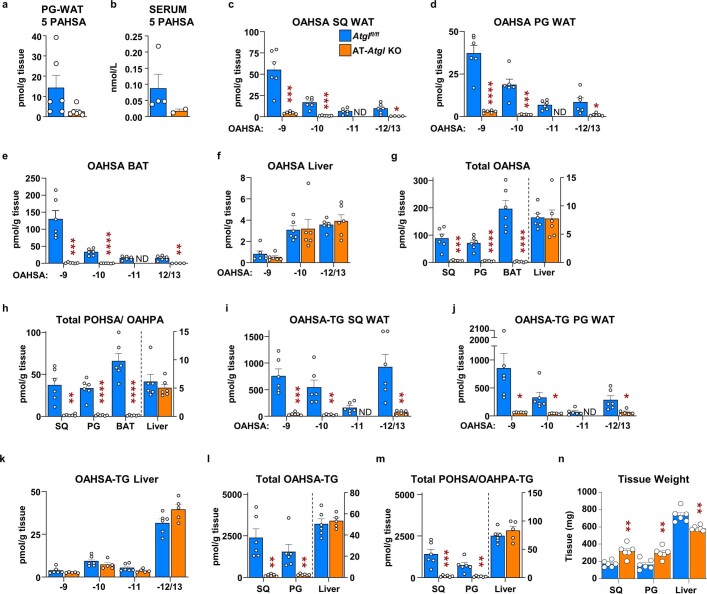
(related to Figure 3): PG WAT and serum 5-PAHSA, free and TG-esterified OAHSA and POHSA/OAHPA levels are lower in adipose tissue of AT-*Atgl*-KO mice. *Atgl*-KO and littermates *Atgl*^*flfl*^ female mice (10-11 weeks old) were sacrificed in the ad-lib fed state for experiments in panels (**a-n)**. Endogenous 5-PAHSA levels in **(a)** PG WAT and **(b)** serum. Endogenous OAHSA isomers levels in **(c)** SQ WAT, **(d)** PG WAT, **(e)** BAT and **(f)** liver. Tissue levels of **(g)** total OAHSA and **(h)** total POHSA/OAHPA. TG-esterified OAHSA isomer levels in **(i)** SQ WAT, **(j)** PG WAT and **(k)** liver. Tissue levels of **(l)** total TG-esterified OAHSA and **(m)** total TG-esterified POHSA/OAHPA. **(n)** Adipose and liver tissue weights. (n = 5 per genotype for serum samples and 6 for all other tissues, mean ± SEM), *P < 0.05, **P < 0.01, ***P < 0.001, ****P < 0.0001 vs. FL/FL mice, t-test, two-tailed (**a-n)**. Fewer than 5 samples for panel **b** or 6 samples for panels **a** and **c-n** indicates the other samples were below the limit of quantification for our MS method. ND means not detected. Source data

To determine the physiological relevance of ATGL as a FAHFA biosynthetic enzyme in vivo, we studied mice lacking ATGL in adipose tissue (AT-*Atgl*-KO mice)^39^. Adipose-tissue-specific deletion of *Atgl* led to a marked decrease in 9-, 10-, 11- and 12/13- PAHSA regioisomers and a 78–94% decrease in total PAHSA levels in SQ WAT, perigonadal (PG) WAT and BAT (Fig. 3h–k) consistent with data in global-*Atgl*-KO mice^28^. By contrast, liver PAHSA levels are similar to those of control *Atgl*^*fl/fl*^ mice (Fig. 3h, l). Serum 9- and 12/13-PAHSAs are lower and total serum PAHSA levels are decreased by 69% in AT-*Atgl*-KO mice (Fig. 3h, m). Levels of 5-PAHSA tended to decrease in PG WAT and serum in AT-*Atgl*-KO mice (Extended Data Fig. 5a, b). These results suggest that adipose tissue is a major contributor to circulating PAHSA levels. Similarly, OAHSA and POHSA/OAHPA levels are also decreased in AT-*Atgl*-KO SQ WAT, PG WAT and BAT by 92–98% but not in liver (Extended Data Fig. 5c–h). We also found that FAHFA-TG levels were 80–94% lower in WAT and BAT from AT-*Atgl*-KO mice, but unchanged in liver (Fig. 3n–q and Extended Data Fig. 5i–m). The different magnitude of reduction among specific FAHFA family members and regioisomers in adipose tissue of AT-*Atgl*-KO mice suggests that ATGL biosynthetic activity may be greater for certain regioisomers and family members. Triglyceride content per gram of tissue is increased in SQ WAT (by 43%) and BAT (by 172%) but not in PG WAT of AT-*Atgl*-KO mice compared with those of *Atgl*^*fl/fl*^ mice (Fig. 3r). Consistent with the well-known function of ATGL as a lipase, SQ and PG WAT weight is increased in young AT-*Atgl*-KO mice (Extended Data Fig. 5n) as shown before^26,39^. Thus, the total amount of triglyceride content in the fat depots in AT-*Atgl*-KO mice is increased as expected in the absence of ATGL-mediated hydrolysis. The fact that an important substrate for FAHFA biosynthesis, triglycerides, is increased but levels of FAHFA and FAHFA-TGs are nearly undetectable strongly supports the conclusion that ATGL is a critical FAHFA biosynthetic enzyme in WAT and BAT. Triglyceride levels and tissue weight are decreased in AT-*Atgl*-KO liver (Fig. 3r and Extended Data Fig. 5n), consistent with the literature^26,39^. Together, these data underscore the physiological importance of ATGL transacylase activity to regulate adipose FAHFA levels. This is a previously unknown function for this enzyme.

To provide direct evidence that ATGL catalyses FAHFA biosynthesis in vivo, we injected AT-*Atgl*-KO mice with D_20_-9-HSA (5 mg kg^−1^, intraperitoneally) and measured the incorporation of this lipid into tissue FAHFAs as a direct measure of endogenous biosynthesis. This resulted in 90% lower 9-PA-D_20_HSA levels in PG WAT of AT-*Atgl*-KO compared to *Atgl*^*fl/fl*^ mice (Fig. 3s). Percentage enrichment of 9-PA-D_20_HSA (that is, 9-PA-D_20_HSA divided by the sum of endogenous 9-PAHSA + 9-PA -D_20_HSA) is also markedly decreased, indicating that reduced FAHFA biosynthesis is responsible for lower endogenous FAHFA levels in PG WAT of AT-*Atgl*-KO mice (Fig. 3t). Newly synthesized 9-PA-D_20_HSA is not detectable in liver or serum of either genotype under these conditions (Fig. 3s). Nascent 9-PA-D_20_HSA-TGs were detected in WAT only in *Atgl*^*fl/fl*^ mice (Fig. 3u). Levels of endogenous HSA substrates and enrichment of D_20_-9-HSA are similar in the WAT of *Atgl*^*fl/fl*^ and AT-*Atgl*-KO mice (Fig. 3v). The reduced FAHFA biosynthesis is cell autonomous, as it is also markedly reduced in AT-*Atgl*-KO adipocytes, resulting in 60–84% lower levels of nascent FAHFAs (Fig. 2i–k). These data prove that ATGL in adipose tissue is critical for FAHFA biosynthesis in vivo and lack of this activity results in lower endogenous adipose tissue and serum FAHFA levels in AT-*Atgl*-KO mice.

**Extended Data Fig. 6:**
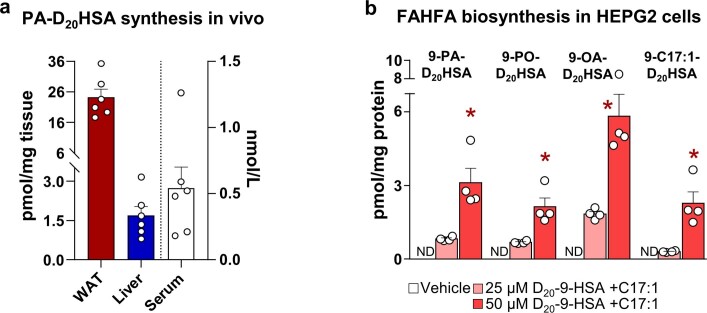
Newly synthesized FAHFAs are present in mouse liver and are synthesized by human hepatocytes. **(a)** WT mice were injected with FAHFA substrate D_20_-9-HSA (25mg/kg) IP and sacrificed 4hrs later to collect serum and tissues. WAT, liver and serum levels of newly synthesized 9-PA-D_20_HSA. (n = 6 mice per condition, mean±SEM) **(b)** 9-FAHFA biosynthesis in intact HEPG2 cells (human hepatocyte) incubated with D_20_-9-HSA, C17:1 or Vehicle (0.05% DMSO) for four hrs. (n = 4 wells per condition, mean±SEM), *P < 0.008 vs. 25µM substrate group t-test, two-tailed. ND means not detected. Source data

The lack of detection of newly synthesized FAHFAs and FAHFA-TGs in liver may be because they are below the detection limit for our liquid chromatography tandem mass spectrometry (LC–MS/MS) method. Endogenous FAHFA and FAHFA-TG levels in *Atgl*^*fl/fl*^ mice are lower in the liver than in adipose tissue^19^ (Fig. 3h and Extended Data Fig. 5g, h, l, m). Experiments with higher doses of D_20_-9-HSA (10–25 mg kg^−1^; Extended Data Fig. 6a) or longer duration (8 h) after injecting D_20_-9-HSA, revealed newly synthesized FAHFAs in the liver as well as the adipose tissue and serum. However, these FAHFAs could be synthesized in the liver or in other tissues and taken up by the liver. Human hepatocytes (HepG2 cells) can synthesize FAHFAs (Extended Data Fig. 6b), indicating that liver is capable of synthesizing FAHFAs.

## DGAT drives ATGL-mediated FAHFA biosynthesis

**Fig. 4:**
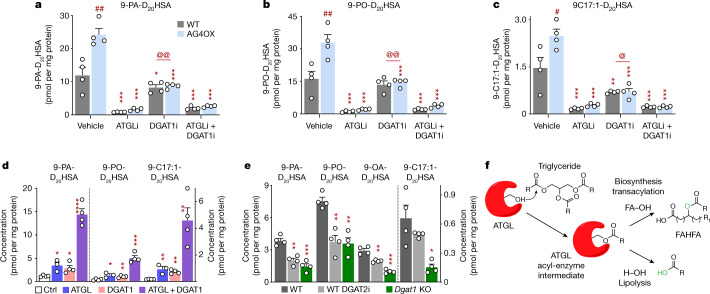
DGATs have an upstream role in ATGL-mediated FAHFA biosynthesis. Cells were incubated with C17:1 FA and D_20_-9-HSA (20 µM (**a**–**c**) or 25 µM (**d**, **e**) each) for 4 h. **a**–**c**, Biosynthesis of 9-PA-D_20_HSA (**a**), 9-PO-D_20_HSA (**b**) and 9-C17:1-D_20_HSA (**c**) in WT and AG4OX SVF adipocytes with or without ATGL inhibitor (10 µM) and DGAT1 inhibitor (DGAT1i, 20 µM) or 0.2% DMSO (vehicle). *n* = 4 wells per condition; mean ± s.e.m. *P < 0.05, **P < 0.001, ***P < 0.0001 versus vehicle, same genotype, #*P* <  0.001, ##*P* < 0.0001 versus WT, same treatment, @*P* < 0.006, @@*P* < 0.0001 versus atglistatin alone (two-way ANOVA). **d**, Biosynthesis of 9-PA-D_20_HSA, 9-PO-D_20_HSA and 9-C17:1-D_20_HSA in HEK293T cells transfected with control vector (Ctrl), ATGL and DGAT1 plasmids alone or together. *n* = 4 wells per condition except ATGL group *n* = 3; mean ± s.e.m. *P < 0.05, **P < 0.01, ***P < 0.001, ****P < 0.001 versus control vector *t*-test, two-tailed. These data are representative of three independent experiments. **e**, Biosynthesis of 9-PA-D_20_HSA, 9-PO-D_20_HSA, 9-OA-D_20_HSA and 9-C17:1-D_20_HSA in WT SVF adipocytes with or without DGAT2 inhibitor (DGAT2i, 20 µM) and *Dgat1*-KO SVF adipocytes. *n* = 4 wells per condition. Mean ± s.e.m. *P < 0.01, **P < 0.001, ***P < 0.0001 versus WT (one-way ANOVA). **f**, Model for ATGL transacylation activity synthesizing FAHFAs. Canonical ATGL lipase activity transfers an acyl chain of TG to a water molecule (H_2_O) thereby releasing a FA. In the presence of TG and HFA, ATGL transacylation activity transfers an acyl chain of TG to HFA, synthesizing FAHFA. Our data show that the lipase activity of ATGL (Fig. 3e) is much higher than its biosynthetic activity (Fig. 3d) in vitro.

**Extended Data Fig. 7:**
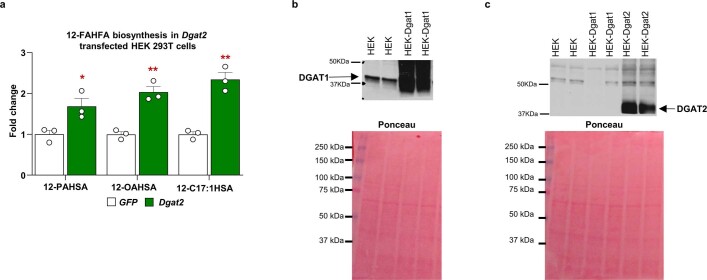
DGAT2 overexpression increases 12-FAHFA biosynthesis in HEK293T cells. **(a)** 12-PAHSA, 12-OAHSA and 12-C17:1HSA biosynthesis in DGAT2-overexpressing HEK293T cells incubated with C17:1 and 12-HSA (25µM each) for 2hrs. n = 3 wells per condition, mean±SEM *P<0.03, **P<0.025 vs GFP group, t-test, two tailed. Representative of 3 independent experiments. Western blot confirming overexpression of DGAT1 **(b)** and DGAT2 **(c)** in HEK 293T cells.

In the current model, the addition of C17:1 FA would require C17:1 FA to be esterified into a triglyceride before transfer to HFA by ATGL. DGAT1 and DGAT2 are important for FA esterification into triglycerides^40^. DGAT1 inhibition resulted in a 30–71% decrease in FAHFA production (Fig. 4a–c), consistent with incorporation of FAs into TGs before they are transferred to HFAs to produce FAHFAs. Inhibition of both DGAT1 and ATGL or ATGL alone led to an 85–91% decrease in FAHFA biosynthesis (Fig. 4a–c). Furthermore, transfection of HEK293T cells with combinations of ATGL and DGAT1 demonstrates that these enzymes can work in tandem to drive FAHFA biosynthesis. Levels of nascent FAHFAs are increased 2.5–6-fold in cells transfected with ATGL or DGAT1 compared to control vector-transfected cells (Fig. 4d). Transfection with both DGAT1 and ATGL resulted in a synergistic increase in production of 9-PA-D_20_HSA (by 12-fold), 9-PO-D_20_HSA (by 12-fold) and 9-C17:1-D_20_HSA (by 27-fold) compared to control cells (Fig. 4d). These effects are not limited to DGAT1. DGAT2 overexpression increased synthesis of 12-PAHSA, 12-OAHSA and 12-C17:1HSA (Extended Data Fig. 7a). Transient transfection of HEK293T cells results in robust overexpression of ATGL (Extended Data Fig. 2a), DGAT1 and DGAT2 enzymes (Extended Data Fig. 7b, c). Furthermore, either DGAT2 inhibition or *Dgat1* KO also decreased FAHFA biosynthesis (Fig. 4e). These results are consistent with DGAT synthesis of TGs driving FAHFA biosynthesis through ATGL transacylation activity.

## Discussion

We identified a biosynthetic enzyme for FAHFAs, ATGL, which we demonstrate catalyses the esterification of HFA and FA using TG, and to a lesser extent diglyceride, as the FA donor. We show that ATGL catalyzes the major FAHFA biosynthesis pathway in adipocytes and that ATGL inhibition decreases nascent FAHFAs and FAHFA-TGs without changing FA and glycerolipids levels. FAHFA and FAHFA-TG levels and biosynthesis are markedly reduced in WAT of AT-*Atgl*-KO mice. This results from loss of ATGL transacylation activity and not lipase activity, which would increase FAHFA-TGs. The observation that fasting and cold exposure increase FAHFA and FAHFA-TG levels in WAT of WT mice but not in global-*Atgl*-KO mice^1,28^ further underscores that ATGL-catalysed FAHFA biosynthesis dominates over any potential hydrolytic activity of ATGL^20^ or other lipases towards FAHFAs^20^ or FAHFA-TGs^20^. Whereas ATGL can shuffle FAHFAs as acyl chains in TGs^20^, there is no evidence before our current data that ATGL can esterify HFAs with FAs (from TGs) to make FAHFAs. This is a paradigm shift in terms of the function of ATGL. Our observation that FAHFAs are synthesized before they are esterified into TGs is further evidence for the importance of ATGL transacylation activity. Mechanistically, both transacylase and lipase activities of ATGL require the formation of an acyl-enzyme intermediate, and this is followed by transfer of the acyl chain to a hydroxy group on an HFA (FAHFA biosynthesis) or to water (lipolysis; Fig. 4f).

Insulin-resistant states such as dietary obesity in mice and humans are associated with reduced adipose and/or serum PAHSA levels, and PAHSA administration to insulin-resistant mice enhances insulin sensitivity^1,5,7^. One might expect AT-*Atgl*-KO mice to be insulin resistant because PAHSA levels are low. However, adipose ATGL also has a crucial role in regulating FA availability to multiple tissues. For example, AT-*Atgl*-KO mice have reduced lipolysis that limits FA flux to the liver, resulting in decreased substrate availability for hepatic glucose production^39,40^. Similarly, FA availability to muscle and other tissues is limited, which can result in utilization of liver glycogen stores and hypoglycemia^26^. The fact that both adipose-specific-ATGL overexpression^41^ and deletion improve insulin sensitivity in mice^26^ reflects the complexity of the multiple metabolic effects of adipose ATGL.

Humans who are obese and resistant to insulin have lower ATGL levels in WAT compared to people who are obese and sensitive to insulin^42^, suggesting that ATGL may contribute to the regulation of FAHFAs in WAT and serum in people^1^. Thus, understanding the regulation of ATGL transacylase activity could lead to strategies to increase the levels of these beneficial lipids in disease conditions.

## Methods

### Reagents

All reagents were purchased from Thermo Fisher unless specified otherwise. Collagenase D (11088858001) and dispase II (04942078001) were purchased from Roche. Ammonium acetate (372331), ammonium hydroxide (338818), MAFP (M2939) and C17:1 FA (H8896) were purchased from Sigma. Enzyme inhibitors were as follows: atglistatin (ATGL inhibitor), A 922500 (DGAT1 inhibitor), TSI-01 (lysophosphatidylcholine acyltransferase inhibitor), ML-348 (LYPLA1 inhibitor), ML-349 (LYPLA2 inhibitor) and etomoxir (carnitine palmitoyltransferase 1 inhibitor) were purchased from Cayman. PF-06424439 (DGAT2 inhibitor) was purchased from Sigma. Acyl donors were as follows: TG(18:1) (95%, Cayman 26871), TG(18:1) (99%, Sigma T7140), TG(16:1) (98%, Sigma T2630), TG(18:2) (98%, Sigma T9517), TG(16:0/18:1/18:1) (98%, Cayman 28559), TG(17:1) (≥98%, Cayman, 26996), DG(18:1/18:1/0:0) (≥97%, Sigma D0138), PC(18:1/18:1) (Cayman 15098) and oleoyl-CoA (FA-CoA, Avanti 870719) were purchased from the indicated companies. 9-has (ref. ^43^), D_20_-9-HSA_,_ FP-alkyne, [^13^C_16_]9-PAHSA (ref. ^1^) and 9-C_9_-HSA were synthesized by the laboratory of D.S. Chemical synthesis is described in the Supplementary Methods. D_31_-palmitic acid was purchased from Cayman (16497). HEK293T, HPEG2 and 3T3L1 cell lines were purchased from ATCC and tested for mycoplasma contamination. WT and catalytically dead mouse ATGL plasmids^37^ and immunopurified CGI-58 enzyme were described before^44,45^. Full-length ATGL was expressed with an N-terminal 6xHis-tag in Expi293F cells (Thermo Fisher Scientific) and purified by affinity chromatography using an Akta pure chromatography system (Cytiva). Mouse ATGL-288 (amino acids 1–288) and ATGL-288(S47A) were immunopurified using the following method: mouse ATGL-288 and mouse ATGL-288(S47A) with an N-terminal 6×His-tag and a C-terminal Strep-tag II were expressed in *Escherichia coli* Arctic Express cells at 10 °C and purified by two-step affinity chromatography using an Äkta avant 25 chromatography system (Cytiva)^38^. We obtained highly purified WT ATGL (His–mAT288–Strep) and catalytically dead mutant (His–mAT288(S47A)–Strep) ATGL as assessed by Coomassie staining of SDS–polyacrylamide gel electrophoresis gels (Extended Data Fig. 4a, b). Mouse DGAT1/2 plasmid^46^, DGAT1 and DGAT2 antibodies^47^ and *Dgat1*-KO^48^ mice were a gift from R. V. Farese’s laboratory. Solvents for LC–MS were purchased from Honeywell Burdick & Jackson. Human SVF pre-adipocytes and SQ tissue biopsies were provided by the Adipose Tissue Biology and Nutrient Metabolism Core (Boston Nutrition Obesity Research Center). The samples were completely de-identified and were provided in a manner that does not link the samples to any private protected information. All adipose tissue donors signed an informed consent form approved by the Boston Medical Center and Boston University Medical Campus Institutional Review Board. The use of de-identified human samples for this study was approved by the Institutional Review Board at Beth Israel Deaconess Medical Center.

### Cell culture and differentiation

#### 3T3-L1 and SVF cell differentiation

3T3-L1 fibroblasts were cultured in high-glucose DMEM, supplemented with 10% FBS and antibiotic–antimycotic, at 37 °C and 5% CO_2_. Pre-adipocytes from the SVF of the SQ WAT were isolated from 9–10-week-old mice. Briefly, both fat pads were minced using scissors and digested in 5 mg ml^−1^ collagenase D, 2 U ml^−1^ dispase II and 10 mM CaCl_2_. SVF cells were then separated from adipocytes using 40-µm cell strainers and seeded on a 10-cm plate in DMEM F12 GlutaMAX supplemented with 15% FBS and 1% antibiotic–antimycotic. After 24 h, unattached cells were removed with PBS washing. SVF pre-adipocytes were cultured until 80–90% confluency. Cells from multiple mice were pooled before differentiation, so each treatment condition was performed on replicate wells from 6–9 mice.

For adipocyte differentiation, SVFs and 3T3-L1 cells were seeded in 12-well plates and grown to confluency. Differentiation medium (DMEM F12 GlutaMAX, 10% FBS, 1% antibiotic–antimycotic, 4 µg ml^−1^ bovine insulin, 1 µM dexamethasone, 0.5 mM 3-isobutyl-1-methylxanthine and 2 µM rosiglitazone) was added to SVFs the day after confluency, and to 3T3-L1 cells 3 days after confluency. SVFs and 3T3-L1 were cultured for 3 days in differentiation medium. Cells were then cultured in a post-differentiation medium containing 10% FBS, 1% antibiotic–antimycotic and 4 µg ml^−1^ bovine insulin for 7–8 days. Human SVF pre-adipocytes were cultured and differentiated as described previously^49^. Uniform lipid droplet formation among cells was confirmed before FAHFA biosynthesis experiments through visual inspection under a microscope and/or oil red O staining.

#### Transfections in HEK293T cells

Cells were cultured in high-glucose DMEM, supplemented with 10% FBS and 1% antibiotic–antimycotic, at 37 °C and 5% CO_2_. For FAHFA biosynthesis experiments, HEK293T cells were seeded as 200,000 cells per well on 6-well plates (Primaria). After 18–20 h, cells were transiently transfected with 1.5 µg per well WT ATGL, ATGL(S47A) mutant, DGAT1, DGAT2, GFP or control vector plasmid using Lipofectamine 2000. FAHFA biosynthesis experiments were performed in intact cells 48 h after transfections.

### Intact cell FAHFA biosynthesis assays

Transiently transfected HEK293T cells, differentiated mouse and human adipocytes or HEPG2 cells (human hepatoma) were incubated with 9-HSA or D_20_-9-HSA and C17:1 FA in phenol-free medium containing 1% FBS for 2 h (Fig. 1 and Extended Data Fig. 1) or 4 h (Figs. 2 and 4 and Extended Data Figs. 2, 3 and 6) at 37 °C. The specific concentration used for each experiment is indicated in the figure legends. For MAFP, FP-alkyne and enzyme inhibitor studies, adipocytes were pre-incubated with these compounds for 1 h. Adipocytes were preincubated for 1 h with the following inhibitors: atglistatin (ATGL inhibitor, 10 µM), TSI-01 (lysophosphatidylcholine acyltransferase inhibitor, 25 µM), ML-348 (LYPLA1 inhibitor, 10 µM), ML-349 (LYPLA2 inhibitor, 10 µM) and etomoxir (carnitine palmitoyltransferase 1 inhibitor, 10 µM) for experiments in Fig. 2a, b. Adipocytes were co-incubated with FA and HFA substrates, atglistatin (10 µM), A 922500 (DGAT1 inhibitor, 20 µM) and PF-06424439 (DGAT2 inhibitor, 20 µM) for experiments in Figs. 2c–l and 4 and Extended Data Fig. 2 and 3. After incubation with FAs and HFAs, cells were washed with ice-cold PBS once and collected in sterile 550 µl PBS. Cells were stored at −80 °C until lipid extractions. Protein concentrations were determined using the bicinchoninic acid assay at the time of lipid extraction.

### Activity-based proteomics sample preparation

WT and AG4OX SVF adipocytes were pretreated with 5 µM FP-alkyne for 1 h and then co-incubated with 9-HSA for 2 h. Cells were then washed with ice-cold PBS twice, scraped, sonicated to obtain cell lysates and stored at −80 °C.

Click chemistry and biotinylated protein enrichment was performed as described previously^50^. Alkyne-labelled proteomes (1.5 mg ml^−1^ in 1 ml PBS) were incubated with biotin-N_3_ (100 μM), TCEP (1 mM), TBTA (100 μM) and CuSO_4_ (1 mM) for 1 h at room temperature while rotating. After labelling, the proteomes were denatured and precipitated using 2 ml cold methanol, and protein pellets were washed with 1 ml of 1:1 cold methanol/CHCl_3_ (twice), sonicated in 2.5 ml 4:1 methanol/CHCl_3_ and resuspended in 1 ml PBS with 0.5% SDS. The biotinylated proteins were enriched with PBS-washed avidin–agarose beads (80 µl; Thermo Scientific, catalogue number 20357) by rotating at room temperature for 2 h. The beads were then washed sequentially with 0.5% SDS in PBS (four times). To elute proteins, beads were incubated in 4% SDS, 5% glycerol, 200 mM 2-mercaptoethanol and 100 mM Tris pH 6.8 (160 µl) at 95 °C for 10 min. The supernatant sample was collected.

Proteins were precipitated using methanol–chloroform. Dried pellets were dissolved in 8 M urea/100 mM TEAB, pH 8.5, reduced with 5 mM tris(2-carboxyethyl) phosphine hydrochloride (TCEP), and alkylated with 50 mM chloroacetamide. Samples were diluted to 2 M urea/100 mM TEAB, and proteins were then trypsin digested overnight at 37 °C.

### Proteomics LC–MS and data analysis

The digested samples were analysed on an Orbitrap Fusion mass spectrometer (Thermo). Samples were injected directly onto a 25-cm, 100-μm ID column packed with BEH 1.7-μm C18 resin (Waters). Samples were separated at a flow rate of 300 nl min^−1^ on an nLC 1200 (Thermo). The LC solvents were as follows: buffer A, 0.1% formic acid in water; buffer B, 90% acetonitrile/0.1% formic acid. A typical LC run was 240 min long with a binary gradient consisting of the following steps: 1–25% buffer B over 180 min, 25–40% B over 40 min, 40–90% B over 10 min, 90% B for 10 min. The column was re-equilibrated with buffer A before the injection of sample. Peptides were eluted directly from the tip of the column and nanosprayed directly into the mass spectrometer by application of 2.5 kV voltage at the back of the column. The mass spectrometer was operated in a data-dependent mode. Full MS1 scans were collected in the Orbitrap at 120,000 resolution. The cycle time was set to 3 s, and within this 3 s the most abundant ions per scan were selected for collision-induced dissociation MS/MS in the ion trap. Monoisotopic precursor selection was enabled and dynamic exclusion was used with an exclusion duration of 5 s.

Protein and peptide identification were performed with Integrated Proteomics Pipeline (IP2, Integrated Proteomics Applications). Tandem mass spectra were extracted from raw files using RawConverter^51^ and searched with ProLuCID^52^ against the UniProt mouse database. The search space included all fully tryptic and half-tryptic peptide candidates. Carbamidomethylation on cysteine was considered as a static modification. Data were searched with a 50-ppm precursor ion tolerance and a 600-ppm fragment ion tolerance. Identified proteins were filtered to a 10-ppm precursor ion tolerance using DTASelect^53^ utilizing a target–decoy database search strategy to control the false discovery rate to 1% at the protein level^54^.

### Western blotting

Frozen adipose tissue (human or mouse) and HEK293T cells were dounce homogenized on ice in buffer A (0.25 M sucrose, 1 mM EDTA, 1 mM dithiothreitol, 20 µg ml^−1^ leupeptin, 2 µg ml^−1^ antipain and 1 µg ml^−1^ pepstatin, pH 7.0). Lysates were passed through a 26-G or 30-G needle attached to a 1-cc syringe 30 times to break open the cells. Samples were sonicated in a 550 Sonic Dismembrator Cup Horn (Fisher Scientific) filled with ice-cold water for 5 min. Homogenates were centrifuged to remove nuclei and unbroken cells (1,000*g*, 2 °C, 15 min). Protein concentrations were determined using the reducing reagent bicinchoninic acid assay (Thermo Fisher). Fifty micrograms of proteins were separated by SDS– polyacrylamide gel electrophoresis (7.5% Bio-Rad TGX gels) and transferred to nitrocellulose membranes. Membranes were stained using Revert 700 (Licor) to visualize and quantify total proteins on the blots. Blots were blocked with 5% milk in TBS for 30 min. Membranes were probed with antibodies to ATGL (Cell Signaling number 2439, 30A4, 1:2,000), DGAT1 and DGAT2 (gift from R. V. Farese’s laboratory, 1:1,000)^47^ and GAPDH (Cell Signaling number 2118, 14C10, 1:5,000). IR Dye 800CW anti-rabbit and anti-mouse secondary antibodies (LI-COR,1:15,000) were used as secondary antibodies for visualization. The uncropped and unprocessed scans are shown in Supplementary Figs. 1–3. Band intensities were quantified using Image Studio Lite software.

### FAHFA biosynthesis assay with affinity-purified ATGL and CGI-58

The in vitro FAHFA biosynthesis assay was adapted from a previously described ATGL activity assay^55^. For the preparation of the substrate for each reaction, 15 µg PC/phosphatidylinositol 3:1 w/w) was added to the substrates in a glass tube. Organic solvent was evaporated off under a N_2_ stream. The mixture was emulsified by sonication (550 Sonic Dismembrator, Fisher Scientific) in 100 µl of 0.1 M potassium phosphate, 5% BSA pH 7.0. For the acyl donor screen experiment, PC/phosphatidylinositol was omitted from the substrate preparation. Final acyl donor concentrations were 850 µM for TG(18:1), TG(17:1), TG(16:1), TG(18:2), TG(16:0/18:1/18:1), C17:1 FFA, DG(18:1/18:1/0:0), PC(18:1/18:1) and 100 µM for oleoyl-CoA (18:1-CoA). An 800 µM concentration (final) of 9-HSA or D_20-_9-HSA was the acyl acceptor. (See purity of each substrate above.) For the assay, 10 µg immunopurified mouse enzymes WT full-length ATGL, WT ATGL-288 or ATGL-288(S47A) alone or with the cofactor CGI-58 were constituted in 100 µl reaction buffer (0.25 M sucrose, 1 mM EDTA, 1 mM dithiothreitol, 20 µg ml^−1^ leupeptin, 2 µg ml^−1^ antipain and 1 µg ml^−1^ pepstatin, pH 7). For atglistatin inhibition experiments (Fig. 3a, right), full-length ATGL enzyme was preincubated with the indicated doses of atglistatin or 0.5% dimethylsulphoxide (DMSO) for 15 min on a shaking thermo mixer at 37 °C. To initiate the FAHFA biosynthesis reaction, substrate emulsion was added to the enzymes. For each assay, 100 µl substrate with 100 µl reaction buffer with or without enzymes was incubated for 60 min at 37 °C while shaking. The reaction was stopped by addition of 500 µl methanol. Samples were stored at −80 °C for FAHFA extraction.

Using 99% pure TG(18:1) substrate from Sigma, compared to 95% pure TG(18:1), from Cayman resulted in a higher rate of 9-OAHSA synthesis in our in vitro biosynthesis experiments. As all comparisons were made within the same experiment, this did not affect the relative values within an experiment. For the IC_50_ calculation in Fig. 3a (right), we used a nonlinear regression log[inhibitor] versus response (four parameters) model and used the mean value of the biosynthesis assay with no enzyme as the bottom constraint.

### Human SQ WAT lysate FAHFA biosynthesis assay

The assay was performed as described above, except 400 µg quantities of human SQ WAT lysates from three different donors were incubated with substrates TG and D_20_-9-HSA for 60 min at 37 °C while shaking.

### Mouse studies

All experimental procedures were approved by the Institutional Animal Care and Use Committee of Beth Israel Deaconess Medical Center and performed following its policies. Mice were housed at the Beth Israel Deaconess Medical Center at 23.3 °C temperature and 40–60% humidity, on ventilated racks (25 ACH) under a 12 h light/12 h dark cycle and fed on chow diet (Lab Diet, 5008). AG4OX mice were generated in our laboratory as previously described^39,56^. AG4OX and control WT female and male mice (FVB background) of 9–10 weeks of age were used for SVF adipocyte preparations. AT-*Atgl*^*fl/fl*^ (C57BL/6J) mice were purchased from Jackson Laboratory (stock number 024278) and crossed to adiponectin-Cre mice (C57BL/6J), a gift from E. D. Rosen^57^ (Beth Israel Deaconess Medical Center) to generate AT-*Atgl*-KO mice. The metabolic phenotype of AT-*Atgl*-KO mice was previously described^39^ and was confirmed in our colony. AT-*Atgl*-KO and littermate *Atgl*^*fl/fl*^ control female mice of 10–11 weeks of age were used for studies of endogenous FAHFA levels in tissues and in vivo measurement of FAHFA biosynthesis. Animals were euthanized by CO_2_ inhalation, blood was collected by cardiac puncture to obtain serum, and tissues were rapidly dissected, flash-frozen in liquid nitrogen and stored at −80 °C.

### FAHFA biosynthesis in vivo

For in vivo experiments, mice were randomly assigned to different groups. The experiment was performed between 08:00 and 13:15. Ad-libitum-fed AT-*Atgl*-KO and *Atgl*^*fl/fl*^ control mice were intraperitoneally injected with 5 mg kg^−1^ D_20_-9-HSA in 49.5% H_2_O/ 0.5% Tween 20, PEG 400/50% vehicle. The volume of the injection was adjusted on the basis of body weight (5 µl g^−1^ body weight; that is, 100 µl for a 20-g mouse). Food was immediately removed. In our experience with this strain of mice, 5-h food removal in the morning does not increase serum non-esterified FA levels (ad libitum fed 0.68 ± 0.03; after 5-h food removal 0.75 ± 0.04 mmol l^−1^, *n* = 7 per group), which is consistent with no significant increase in lipolysis. Injections were staggered by 7 min, so each mouse was euthanized exactly 4 h after substrate injection for tissue and serum collection. Serum, liver and PG WAT lipids were extracted and D_20_-9-HSA incorporation into FAHFAs and TG-esterified FAHFAs was measured by LC–MS. A further biosynthesis study with a D_20_-9-HSA dose of 25 mg kg^−1^ (Extended Data Fig. 6a) was also performed in the same manner in WT mice.

### Lipid extraction

For the measurement of FAHFAs from cultured cells, total lipids were extracted using the modified Bligh–Dyer method^58^. In brief, 1.5 ml of 2:1 chloroform/methanol with the internal standard [^13^C_16_]9-PAHSA (5 pmol) was added to 500 µl of cell suspension in PBS. Samples were vortexed and centrifuged at 2,000*g* for 7 min. The bottom organic phase was transferred into a new vial, and dried under a N_2_ stream.

For tissues (50–75 mg) and serum (100 µl), total lipids were extracted as described above. FAHFAs were enriched using a solid-phase extraction (SPE) column (Hypersep silica 500 mg) as described previously^59^. Briefly, columns were equilibrated with 15 ml hexane, and the samples were then resuspended in 200 µl chloroform and loaded on the SPE column. The neutral lipid fraction containing TGs was eluted with 16 ml of 95:5% hexane/ethyl acetate, and the polar lipid fraction containing non-esterified FAHFAs was eluted using 15 ml ethyl acetate. The FAHFA fractions were dried under a stream of N_2_ and stored at −40 °C until LC–MS/MS analysis. Total TG levels were quantified from the same piece of tissue as FAHFAs. Neutral lipid fractions of SPE eluents were used to quantify total tissue TG levels using the Infinity triglyceride colorimetric assay kit (Thermo, TR22421) as previously described^60^.

TG-esterified FAHFAs were extracted, hydrolysed and enriched by multi-step lipid extractions and SPE fractionations with a mild LiOH hydrolysis method as described elsewhere^27^. Internal standard TG ([^13^C_16_]PAHSA/16:0/16:0) was added at the beginning of sample processing and D_31_-PAHSA internal standard was added after 24-h mild hydrolysis of neutral fractions. The amount of internal standard was adjusted on the basis of the type of tissue or cell. Samples were stored at −40 °C until FAHFAs released by mild hydrolysis (TG-esterified FAHFAs) were measured by LC–MS analysis.

TG-esterified C17:1 and D_20_-9-HSA were extracted in the same manner as TG-esterified FAHFAs for the experiment in Fig. 2l. An internal standard TG(D_9_-16:0/16:0/16:0) (Cayman 30181) was added at the beginning of sample processing and D_31_-16:0 (Cayman 16497) internal standard was added to the samples after mild hydrolysis of neutral fractions to quantify TG-esterified C17:1 and D_20_-9HSA.

### Sample analysis using LC–MS/MS

FAHFA isomers were quantified using an Agilent 6470 Triple Quad LC–MS/MS instrument through multiple reaction monitoring (MRM) in the negative ionization mode as described previously^27^. In brief, cell culture lipid extracts were reconstituted in 50 µl methanol, and tissue extracts were reconstituted in 100 µl methanol. A 7 µl volume of sample was injected onto a UPLC BEH C18 Column (Waters Acquity, 186002352). FAHFA regioisomers from different FAHFA families were resolved using a 93:7 methanol/water with 5 mM ammonium acetate and 0.01% ammonium hydroxide solvent through an isocratic gradient at 0.15 ml min^−1^ flow rate for 45 min. Transitions for targeted FAHFAs are listed in Supplementary Table 1. MS acquisition parameters for tandem MS were: gas temperature = 250 °C, gas flow = 12 l min^−1^, nebulizer = 20 psi, sheath gas temperature = 250 °C, sheath gas flow = 11 l min^−1^. Spray voltage was −1.0 kV.

Each FAHFA regioisomer as well as newly synthesized 9-FA-D_20_HSA levels were quantified by normalizing their peak area (extracted using MassHunter 10.0) to the internal standard [^13^C_16_]9-PAHSA peak area and total protein amount or tissue weight. For the in vivo biosynthesis experiment, enrichment of newly synthesized PAHSA is calculated as:

$$\frac{9-{\rm{PA}}-{{\rm{D}}}_{20}{\rm{HSA}}\times 100}{(9-{\rm{PAHSA}}+9-{\rm{PA}}-{{\rm{D}}}_{20}{\rm{HSA}})}$$

For TG-esterified FAHFA quantifications, levels of FAHFAs hydrolysed from TGs were normalized to levels of [^13^C_16_]PAHSA hydrolysed from the internal standard TG ([^13^C_16_]PAHSA/16:0/16:0). Percentage TG hydrolysis was corrected by the internal standard D_31_-PAHSA as described previously^27^.

### Quantification of FA/HSA and D_20_-9-HSA for in vivo/intact cells and in vitro biosynthesis studies

A 25 mg quantity of PG WAT was homogenized in 1.5:1.5:3 ml PBS/methanol/chloroform with the internal standard 20 pmol ^13^C_9_-9-HSA. Samples were vortexed and centrifuged at 2,000*g* for 7 min. Bottom organic phase was transferred into a new vial, dried under a stream of N_2_.

FFA and HFAs were quantified using an Agilent 6470 Triple Quad LC–MS/MS instrument through MRM in the negative ionization mode. Solvent A was water and solvent B was acetonitrile; solvents contained 5 mM ammonium acetate and 0.01% ammonium hydroxide. A UHPLC BEH C18 column (Waters Acquity, 186002352) was used. FA and HFA were chromatographically separated using a 40-min stepwise gradient. The gradient was held at 20% B at 0 min, increased linearly from 20 to 70% B between 0 and 5 min, increased linearly from 70 to 95% B between 5 and 20 min, held at 95% B between 20 and 25 min, raised to 100% B at 25.1 min, held at 95% B between 25.1 and 30 min, returned to 20% B at 31 min, and held at 20% B. The flow rate was 0.15 ml min^−1^. Samples were resuspended in 500 µl 1:1 chloroform/methanol and 7 µl of the sample was used for the quantifications. Transitions for targeted HFA pseudo MRM are: HSA, 299.3→299.3 (collision energy (CE) = 0); D_20_-9-HSA, 319.4→319.4 (CE = 0); ^13^C_9_-9-HSA, 308.3→308.3 (CE = 0). The parameters for MS were set as follows: gas temperature = 275 °C, gas flow = 12 l min^−1^, nebulizer = 20 psi, sheath gas temperature = 250 °C, sheath gas flow = 11 l min^−1^. Spray voltage was −1.0 kV. Levels of HSA were quantified by normalizing with levels of ^13^C_9_-9-HSA and grams of tissue. The percentage enrichment of D_20_-9-HSA was calculated as:

$$\frac{{{\rm{D}}}_{20}-9-{\rm{HSA}}\times 100}{({\rm{HSA}}+{{\rm{D}}}_{20}-9-{\rm{HSA}})}$$

For quantification of the FFA in the intact cell culture experiment in Fig. 2m and the in vitro biosynthesis study in Fig. 3e, 20 pmol D_31_-PA internal standard was also added at the beginning of the lipid extraction as described above in the section describing the method for FAHFA lipid extraction. Transitions for targeted pseudo MRM are: PA, 255.2→255.3 (CE = 0); PO, 253.2→253.2 (CE = 0); OA, 281.2→281.2 (CE = 0); D_31_-PA, 286.6→286.6 (CE = 0). To quantify ATGL lipase activity in vitro (Fig. 3e), average background (no enzyme) was subtracted from each sample.

### Semi-targeted lipidomics in 3T3-L1 adipocyte lipid extraction and LC–MS

Lipids were extracted using a modified version of the Bligh–Dyer method^58^. In brief, samples were manually shaken in a glass vial (VWR) with 1 ml PBS, 1 ml methanol and 2 ml chloroform containing internal standards ([^13^C_16_]palmitic acid and D7-cholesterol) for 30 s. The resulting mixture was vortexed for 15 s and centrifuged at 2,400*g* for 6 min to induce phase separation. The organic (bottom) layer was retrieved using a Pasteur pipette, dried under a gentle stream of nitrogen, and reconstituted in 2:1 chloroform/methanol for LC–MS analysis.

Lipidomics analysis was performed on a Vanquish HPLC online with a Q-Exactive quadrupole-Orbitrap mass spectrometer equipped with an electrospray ion source (Thermo). Data were acquired in positive and negative ionization modes. Solvent A consisted of 95:5 water/methanol; solvent B was 70:25:5 isopropanol/methanol/water. For positive mode, solvents A and B contained 5 mM ammonium formate with 0.1% formic acid; for negative mode, solvents contained 0.028% ammonium hydroxide. A Bridge (Waters) C8 column (5 μm, 4.6 mm × 50 mm) was used. The gradient was held at 0% B between 0 and 5 min, increased to 20% B at 5.1 min, increased linearly from 20% to 100% B between 5.1 and 55 min, held at 100% B between 55 min and 63 min, returned to 0% B at 63.1 min, and held at 0% B until 70 min. The flow rate was 0.1 ml min^−1^ from 0 to 5 min, 0.3 ml min^−1^ between 5.1 min and 55 min, and 0.4 ml min^−1^ between 55 min and 70 min. The spray voltage was 3.5 kV and 2.5 kV for the positive and negative ionization modes, respectively; the S-lens RF level was 65. The sheath, auxiliary and sweep gases were 50, 10 and 1, respectively. The capillary temperature was 325 °C and the auxiliary gas heater temperature was 200 °C. Data were collected in full MS/dd-MS2 (top 10) mode. The full MS scan was acquired in the range 150–1,500 *m*/*z* with a resolution of 70,000, an automatic gain control (AGC) target of 1 × 10^6^ and a maximum injection time of 100 ms. MS2 was acquired with a resolution of 17,500, a fixed first mass of 50 *m*/*z*, an AGC target of 1 × 10^5^ and a maximum injection time of 200 ms. Stepped normalized collision energies were 20, 30 and 40%. The inclusion list was on, and the instrument was set to pick other ions when idle.

A lipid target list was generated with LipidCreator. Mass accuracy, chromatography retention time and peak integration of all targeted lipids were verified with Skyline^61^. Peak areas were used in data reporting, and data were normalized using internal standards.

### Statistical analyses

Data are shown as individual data points and mean ± s.e.m. The numbers of participants (human WAT lysate study), mice and wells of cells per group are annotated as *n*. Each cell culture biosynthesis experiment was replicated with similar results at least twice. Graphs and statistical analyses were generated using GraphPad Prism 6.0 and 8.0 (GraphPad Software). Specific statistical tests and *n* used for each experiment are listed in the figure legends. Data were analysed using unpaired *t*-tests, two-tailed for single comparisons, *t*-test corrected for Holm–Sidak multiple comparison, one-way ANOVA for multiple comparisons (Holm–Sidak multiple comparison) and two-way ANOVAs for comparison of genotype and treatments (Holm–Sidak multiple comparison) as listed in the figure legends.

### Reporting summary

Further information on research design is available in the Nature Research Reporting Summary linked to this paper.

## Online content

Any methods, additional references, Nature Research reporting summaries, source data, extended data, supplementary information, acknowledgements, peer review information; details of author contributions and competing interests; and statements of data and code availability are available at 10.1038/s41586-022-04787-x.

## Supplementary information

Supplementary Information This file contains Supplementary Figs. 1–3, Table 1 and Methods (related to chemical synthesis).

Reporting Summary

## Data Availability

The datasets and materials generated during the current study are available from the corresponding author on reasonable request. Source data are provided with this paper.

## Source data

Source Data Fig. 3
Source Data Extended Data Fig. 5
Source Data Extended Data Fig. 6
