# Supplementary material for: ATGL is a biosynthetic enzyme for fatty acid esters of hydroxy fatty acids
Source: Nature. 2022 Jun 8;606(7916):968–75. doi: 10.1038/s41586-022-04787-x (PMC9242854; doi:10.1038/s41586-022-04787-x)

**ATGL is a biosynthetic enzyme for fatty acid esters of hydroxy fatty acids**

**Rucha Patel^1a^, Anna Santoro^1^, Dan Tan^2^, Peter Hofer^3^,** **Monika Oberer^3^ Andrew T. Nelson^4^, Srihari Konduri^4^, Dionicio Siegel^4^, Rudolf Zechner^3,5^, Alan Saghatelian^2^, Barbara B. Kahn^1*^**

^1^ Division of Endocrinology, Diabetes and Metabolism, Department of Medicine, Beth Israel Deaconess Medical Center and Harvard Medical School, Boston, Massachusetts 02215, United States

^2^ Clayton Foundation Laboratories for Peptide Biology, Salk Institute for Biological Studies, 10010 North Torrey Pines Road, La Jolla, CA 92037-1002, United States

^3^ Institute of Molecular Biosciences, University of Graz, Heinrichstrasse 31, A-8010 Graz, Austria

^4^ Skaggs School of Pharmacy and Pharmaceutical Sciences, University of California-San Diego, 9500 Gilman Drive, La Jolla, California 92093-0934, United States

^5^ BioTechMed-Graz, 8010 Graz, Austria

^a^ current address: Dicerna Pharmaceuticals, 33 Hayden Ave, Lexington, Massachusetts 02421, United States

***Corresponding Author:** bkahn@bidmc.harvard.edu

**Supplementary content**

Supplementary Figure (1-3 ) and legends --------------------------------------------------------------3

Supplementary Table 1 -----------------------------------------------------------------------------------4

Supplementary Methods related to chemical synthesis-----------------------------------------------5

**Figure 1:** Uncropped blots related to extended data figure 1F. Revert stain for total protein loading (a); ATGL probed on day 1 (b); and ATGL, ACTIN, GAPDH protein expression in SQ WAT of WT and AG4OX mice. GAPDH loading control used for figures in the manuscript was probed on the same blot after ACTTIN and ATGL. Red dotted lines indicate crop positions for extended data figure 1F. Blot was visualized with Licor.

**Figure 2:** Uncropped blots related to extended data figure 2a. Overexpression of ATGL S47A and ATGL WT (a) and GAPDH (b) in HEK293T cells. (c) Ponceau s staining showing total protein loading. GAPDH loading control was probed on the same blot after ATGL. White dotted lines indicate crop positions for extended data figure 2a.

**Figure 3:** Uncropped blots related to extended data Figure 7a-b. Overexpression of DGAT1 (**a**) and DGAT2 (**b**) in HEK293T and COS7 cells. Bottom panel shows Ponceau s (total protein loading) for corresponding blots. Samples were run on two separate gels to investigate DGAT1 and DGAT2. over expression in transfected cells. Each blot was cut in half prior to probing with the respective DGAT primary antibodies. Red dotted lines indicate crop positions for extended data figure 7 a-b.

**Table 1. MRM transitions for endogenous FAHFAs, newly synthesized FA-HFAs and internal standard**

| Transition | Precursor ion | Product ion | CE |
| --- | --- | --- | --- |
| PAHSA_transition1 | 537.5 | 255.2 | 30 |
| PAHSA_transition2 | 537.5 | 281.2 | 25 |
| OAHSA_transition1 | 563.5 | 281.2 | 30 |
| OAHSA_transition2 | 563.5 | 299.3 | 25 |
| POHSA/OAHPA_transition1 | 535.5 | 253.2 | 30 |
| POHSA/OAHPA_transition2 | 535.5 | 281.2 | 25 |
| C17:1HSA_transition1 | 549.5 | 269.2 | 30 |
| C17:1HSA_transition2 | 549.5 | 281.2 | 25 |
| PA-D_20_HSA_transition1 | 557.6 | 255.2 | 30 |
| PA-D_20_HSA _transition2 | 557.6 | 319.4 | 25 |
| OA-D_20_HSA _transition1 | 583.6 | 281.2 | 30 |
| OA-D_20_HSA _transition2 | 583.6 | 319.4 | 25 |
| PO-D_20_HSA_transition1 | 555.6 | 253.2 | 30 |
| PO-D_20_HSA_transition2 | 555.6 | 319.4 | 25 |
| C17:1-D_20_HSA_transition1 | 569.6 | 269.2 | 30 |
| C17:1-D_20_HSA_transition2 | 569.6 | 319.4 | 25 |
| ^13^C_16_-9-PAHSA | 553.5 | 271.3 | 30 |
| D_31_-9-PAHSA | 568.7 | 286.4 | 30 |

Precursor ion is a negative ion of FAHFA [M-H]**^-^**. Transition 1 is a quantifier MRM, product ion is a negative ion fragment fatty acid [FA-H]**^-^**. Transition 2 is a qualifier MRM, product ion is a dehydrated HSA fragment [HSA-H2O]**^-^**. For OAHSA and D_20_HSA incorporated FAHFAs transition 2 is negative ion fragment [HSA-H]**^-^**.Internal standards are shown in red.

**Chemical synthesis details:**

General Information: All reactions were performed in flame- or oven-dried glassware sealed with rubber septa and under a nitrogen atmosphere unless otherwise indicated. Air- and/or moisture-sensitive liquids or solutions were transferred by cannula or syringe. Organic solutions were concentrated by rotary evaporator at 30 millibars with the water bath heated to not more than 50°C unless specified otherwise. Dichloromethane (DCM) was purified with a Pure-Solve MD-5 Solvent Purification System (Innovative Technology). Thin-layer chromatography (TLC) was performed using 0.2 mm commercial silica gel plates (silica gel 60, F254, EMD Chemicals) and visualized with an aqueous potassium permanganate (KMnO_4_) stain. Nuclear Magnetic Resonance (NMR) spectra were recorded on a Varian (^1^H NMR: CDCl_3_ (7.26) at 600 MHz; ^13^C NMR: CDCl_3_ (77.16) at 151 MHz). All spectra were taken in CDCl_3_ with shifts reported in parts per million (ppm) referenced to protium or carbon of the solvent (7.26 or 77.16, respectively). Coupling constants are reported in Hertz (Hz). Data for ^1^H-NMR are reported as follows: chemical shift (ppm, reference to protium; s = single, d = doublet, t = triplet, q = quartet, dd = doublet of doublets, m = multiplet, coupling constant (Hz), and integration). High-Resolution Mass Spectra (HRMS) were acquired on an Agilent 6230 High-Resolution time-of-flight mass spectrometer and reported as m/z for the molecular ion [M+H]^+^.

**Synthesis of 10-bromodecyl hex-5-yn-1-ylcarbamate:** To a stirred solution of 10-bromo-1-decanol (0.2 g, 0.843 mmol, 1.0 equiv) in dry DMF (5.0 mL) under the nitrogen atmosphere was added triethylamine (0.29 mL, 2.108 mmol, 2.5 equiv) and 1,1’-carbonyldiimidazole (0.3 g, 1.855 mmol, 2.2 equiv) simultaneously to the mixture. The reaction mixture was stirred for 12 hours at room temperature. After completion the reaction was quenched with DI water (10 mL) and extracted with EtOAc (3x15 mL). Combined organic layers were washed with saturated brine solution twice, dried over sodium sulfate, filtered, and concentrated under vacuum. The compound was purified by using column chromatography (20% EtOAc: Hexanes), yielding the 10-bromodecyl 1H-imidazole-1-carboxylate (0.19 g, 67%).

R_f_ = 0.4 (silica gel, 70:30 hexanes: EtOAc); ^1^H NMR (600 MHz, CDCl_3_) δ 8.12 (s, 1H), 7.41 (s, 1H), 7.05 (s, 1H), 4.39 (t, *J* = 6.7 Hz, 2H), 3.38 (t, *J* = 6.8 Hz, 2H), 1.82 (dd, *J* = 14.7, 7.1 Hz, 2H), 1.76 (dd, *J* = 14.7, 7.1 Hz, 2H), 1.39 (m, 4H), 1.35 – 1.26 (m, 8H). ^13^C NMR (151 MHz, CDCl_3_) δ 148.84, 137.19, 130.68, 117.20, 68.56, 34.14, 32.84, 29.39, 29.37, 29.16, 28.77, 28.52, 28.18, 25.76. HRMS: *m/z*: calcd for C_14_H_24_BrN_2_O_2_: 331.1016; found 331.1013 [M + H] ^+^.

To a stirred solution of 10-bromodecyl 1H-imidazole-1-carboxylate (110 mg, 0.332 mmol; 1.0 equiv) and Hex-5-yn-1-amine (52 mg, 0.531 mmol, 1.6 equiv) in dry DMF (2.2 ml) was added triethylamine (120 µL, 0.863 mmol, 2.6 equiv) and stirred for 16 hours at RT^1^. Then the solvent was distilled under vacuum. The compound was purified by using column chromatography (15% EtOAc: Hexanes) to yield 10-bromodecyl hex-5-yn-1-ylcarbamate (79 mg, 65%) as a white solid.

R_f_ = 0.5 (silica gel, 80:20 hexanes: EtOAc); ^1^H NMR (600 MHz, CDCl_3_) δ 4.71 (s, 1H), 4.01 (t, *J* = 6.6 Hz, 2H), 3.51 (t, *J* = 6.8 Hz, 2H), 3.18 (dd, *J* = 12.8, 6.3 Hz, 2H), 2.20 (m, 2H), 1.94 (t, *J* = 2.6 Hz, 1H), 1.77 – 1.71 (m, 2H), 1.62 – 1.52 (m, 6H), 1.43 – 1.36 (m, 2H), 1.26 (m, 10H). ^13^C NMR (151 MHz, CDCl_3_) δ 156.89, 84.11, 68.76, 64.99, 45.28, 40.46, 32.70, 29.48, 29.44, 29.31, 29.16, 29.12, 28.93, 26.93, 25.91, 25.61, 18.17. HRMS: *m/z*: calcd for C_17_H_31_BrNO_2_: 360.1533; found 360.1531 [M + H]^+^.

**10-(diethoxyphosphoryl) decyl hex-5-yn-1-ylcarbamate:** A stirred solution of 10-bromodecyl hex-5-yn-1-ylcarbamate (65 mg, 0.180 mmol, 1.0 equiv) in triethyl phosphite (0.19 mL, 1.082 mmol, 6 equiv) was heated to 155°C and stirred for 12 hours. Then the reaction was then cooled to 90°C and concentrated under vacuum completely. The compound was purified using column chromatography by eluting with (5% MeOH: DCM) to afford 10-(diethoxyphosphoryl) decyl hex-5-yn-1-ylcarbamate (34 mg, 45%) as a colorless oil.

R_f_ = 0.6 (silica gel, 90:10 DCM: MeOH); ^1^H NMR (600 MHz, CDCl_3_) δ 4.93 (s, 1H), 4.07 – 3.91 (m, 6H), 3.11 (dd, *J* = 12.6, 6.2 Hz, 2H), 2.14 (m, 2H), 1.89 (t, *J* = 2.3 Hz, 1H), 1.67 – 1.60 (m, 2H), 1.57 – 1.46 (m, 8H), 1.30 – 1.17 (m, 18H). ^13^C NMR (151 MHz, CDCl_3_) δ 156.86, 84.01, 68.64, 64.79, 61.37, 61.32, 40.33, 30.59, 30.48, 29.39, 29.19, 29.03, 26.06, 25.79, 25.52, 25.13, 22.35, 22.31, 18.06, 16.48, 16.44. HRMS: *m/z*: calcd for C_21_H_41_NO_5_P: 418.2717; found 418.2717 [M + H]^+^.

**10-(ethoxyfluorophosphoryl) decyl hex-5-yn-1-ylcarbamate (FP-alkyne):** To a stirred solution of 10-(diethoxyphosphoryl) decyl hex-5-yn-1-ylcarbamate (30 mg, 0.072) in ethanol (0.7 mL) was added a 2M NaOH (0.4 mL) solution. The reaction mixture was heated to 100℃ and stirred for 16 hours. The reaction was cooled to 23 °C, added water (1 mL), and adjusted the pH to 1-2 by using aq. 1M HCl. The compound was extracted with ethyl acetate (3 X 7 mL organic layers were combined and washed with saturated aq. sodium chloride solution. Dried over anhydrous sodium sulfate, filtered and concentrated under the vacuum. Cooled to RT and used for the next step without further purification. The compound was dissolved in dry DCM (1.0 mL) and cooled to -78℃. DAST (28 µl, 0.216mmol, 3 equiv) was added slowly to the reaction mass and stirred for 5 mins. After that slowly raise the temperature to RT and stirred for 45 mins. The reaction was quenched by adding water (1.5 mL) and extracted the compound with ethyl acetate (3*6 mL). Combined the organic layers and washed with saturated aq. sodium chloride solution. Dried over anhydrous sodium sulfate, filtered and concentrated in vacuum. The compound was purified by using column chromatography by eluting (30% EtOAc: Hexanes) yield FP-alkyne probe (14 mg, 52% over two steps) as white solid.

R_f_ = 0.5 (silica gel, 50:50 hexanes: EtOAc); ^1^H NMR (600 MHz, CDCl_3_) δ 4.72 (s, 1H), 4.28 – 4.19 (m, 2H), 4.01 (t, *J* = 6.5 Hz, 2H), 3.18 (dd, *J* = 12.6, 6.2 Hz, 2H), 2.20 (td, *J* = 6.8, 2.5 Hz, 2H), 1.94 (t, *J* = 2.5 Hz, 1H), 1.89 – 1.83 (m, 2H), 1.66 – 1.54 (m, 8H), 1.39 – 1.34 (m, 5H), 1.26 (m, 10H). ^13^C NMR (151 MHz, CDCl_3_) δ 156.92, 84.13, 68.76, 64.99, 63.15, 63.10, 40.48, 30.41, 29.48, 29.29, 29.13, 29.01, 25.91, 25.62, 24.77, 23.96, 21.94, 18.19, 16.47. HRMS: *m/z*: calcd for C_19_H_36_FNO_4_P: 392.2360; found 392.2356 [M + H]^+^.

**D_20_-Nonadec-1-en-10-ol:** A stirred solution of decan-D_21_-1-ol (200 mg, 1.11 mmol, 1.0 equiv) in anhydrous dichloromethane (20 mL) was cooled to 0°C and after the mixture was stirred for 15 min solid Dess-Martin periodinane (993 mg, 2.34 mmol, 2.1 equiv) as added. The cooling bath was removed and the reaction was stirred for 1 hour at 23 °C. The mixture was then cooled to 0°C and a second portion of solid Dess-Martin periodinane (993 mg, 2.34 mmol, 2.1 equiv) was added in a single portion. The cooling bath was removed and the mixture was stirred at 23°C for 2 hours. The mixture was then diluted with water, extracted with dichloromethane (3 x). The dichloromethane layers were combined, washed with brine, dried over sodium sulfate, filtered, and concentrated under vacuum. The crude aldehyde was purified by silica gel column chromatography by using hexanes: EtOAc (94: 6) to yield D_20_-1-decanal (175 mg, 0.992 mmol, 89%). This was used directly in the next step.

A dried flask equipped with a stir bar was charged with magnesium turnings (110 mg, 4.54 mmol, 4.0 equiv) and flame dried under vacuum, cooled to ambient temperature, flushed with dry nitrogen. Anhydrous THF (5 mL) was added by syringe. Rapid stirring was initiated and 2 mg of solid iodine was added. After 5 minutes neat 9-bromonon-1-ene (116 mg, 0.567 mmol, 0.5 equiv) was added dropwise by a syringe. The mixture was stirred until the Grignard activation (observed the color change). The mixture was stirred for 20 minutes at 23 °C and the remaining 9-bromonon-1-ene (349 mg, 1.7 mmol, 1.5 equiv) was added neat, dropwise. Stirring was continued for 45 minutes after the final addition. The reaction mixture was cooled to 0°C and followed by the dropwise the D_20_-1-decanal (200 mg, 1.13 mmol, 1.0 equiv) in THF (3 mL) by syringe. The cooling bath was removed and the solution was allowed to stir at 23 °C for 15 mins. The reaction was quenched by the addition of sat. aq. NH_4_Cl solution, diluted with water and the mixture was extracted with EtOAc (2 x). The ethyl acetate layers were combined, washed with brine, dried over sodium sulfate, and concentrated under vacuum. The crude alcohol was purified bya column chromatography by using hexanes: EtOAc (90:10) to yield D_20_-nonadec-1-en-10-ol (250 mg, 0.826 mmol 74%).

**R_f_** = 0.6 (silica gel, 15: 85 = EtOAc: Hexane); **^1^H NMR** (600 MHz, CDCl_3_) δ 5.81 (m, 1H), 4.99 (dd, *J* = 17.1, 1.5 Hz, 1H), 4.95 – 4.90 (m, 1H), 2.04 (q, *J* = 7.0 Hz, 2H), 1.45 – 1.36 (m, 5H), 1.30 (s, 8H). **HRMS:** m/z: calcd for C_19_H_18_D_20_O: 303.4251; found 303.4244 [M+H] ^+^.

**D-20-9-hydroxystearic acid:** To a stirred solution of D_20_-nonadec-1-en-10-ol (230 mg, 0.760 mmol, 1 equiv) in dioxane-water (15 mL, 3:1) was added neat 2,6-lutidine (163 mg, 1.52 mmol, 2 equiv), OsO_4_ solution (100 μL, 0.015 mmol, 0.02 equiv, 4% in water), and solid NaIO_4_ (650 mg, 3.04 mmol, 4 equiv). The solution was stirred for 4 hours at 23 °C. After completion the solution was diluted with water and dichloromethane. The dichloromethane layer was separated and the aquesous layer was extracted with dichloromethane (2 x). The dichloromethane layers were combined, washed with brine, dried over sodium sulfate, and concentrated under vacuum. The product was purified by column chromatography by using hexane: EtOAc (80: 20) to yield the corresponding aldehyde (225 mg, 0.739 mmol, 97%). The aldehyde was used directly in the next step.

A solution of the aldehyde (above) (220 mg, 0.725 mmol, 1 equiv) and 2-methyl-2-butene (508 mg, 7.25 mmol, 10 equiv) in tert-butanol (5 mL) was cooled to 0°C. Dropwise addition of sodium phosphate monobasic monohydrate (400 mg, 2.90 mmol, 4 equiv) and sodium chlorite (262 mg, 2.90 mmol, 4 equiv) in water (2 mL) followed. The reaction mixture was stirred for 16 hours at 23 °C (color change was observed). The reaction mixture was cooled to 0°C followed by the additions of sodium bisulfite (452 mg, 4.35 mmol, 6 equiv) solution in water (1.1 mL). Afterwards 2-methyl-2-butene and t-BuOH were removed under vacuum and the mixture was dilute with brine and extracted with ethyl acetate (3 x). The combined the ethyl acetate layers were dried over sodium sulfate, filtered, and concentrated. The carboxylic acid was purified via column chromatography by using EtOAc: Hexane (40: 60) yielding D-20-9-hydroxystearic acid (170 mg, 0.532 mmol, 73%).

**R_f_** = 0.5 (silica gel, 50: 50 = EtOAc: Hexane); **^1^H NMR** (600 MHz, CDCl_3_) δ 2.35 (t, *J* = 7.5 Hz, 2H), 1.67 – 1.60 (m, 2H), 1.42 (m, 3H), 1.36 – 1.24 (m, 8H). **HRMS:** m/z: calcd for C_18_H_16_D_20_O_3_: 319.3847; found 319.3849 [M-H] ^-^.

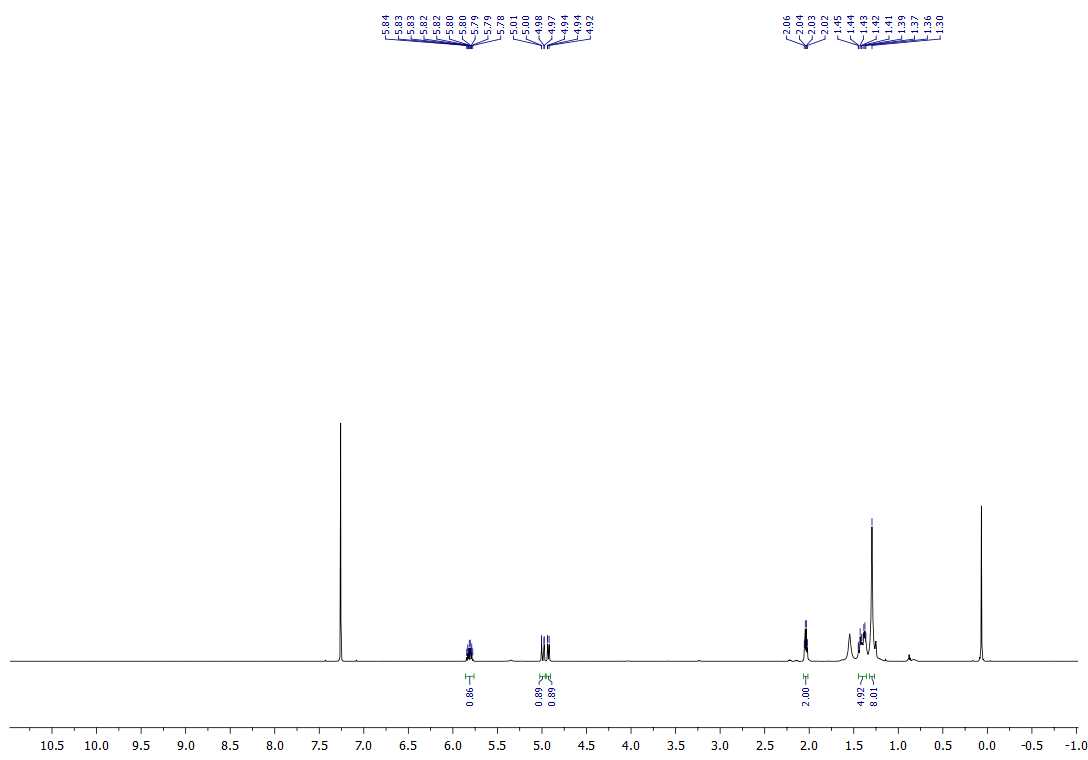

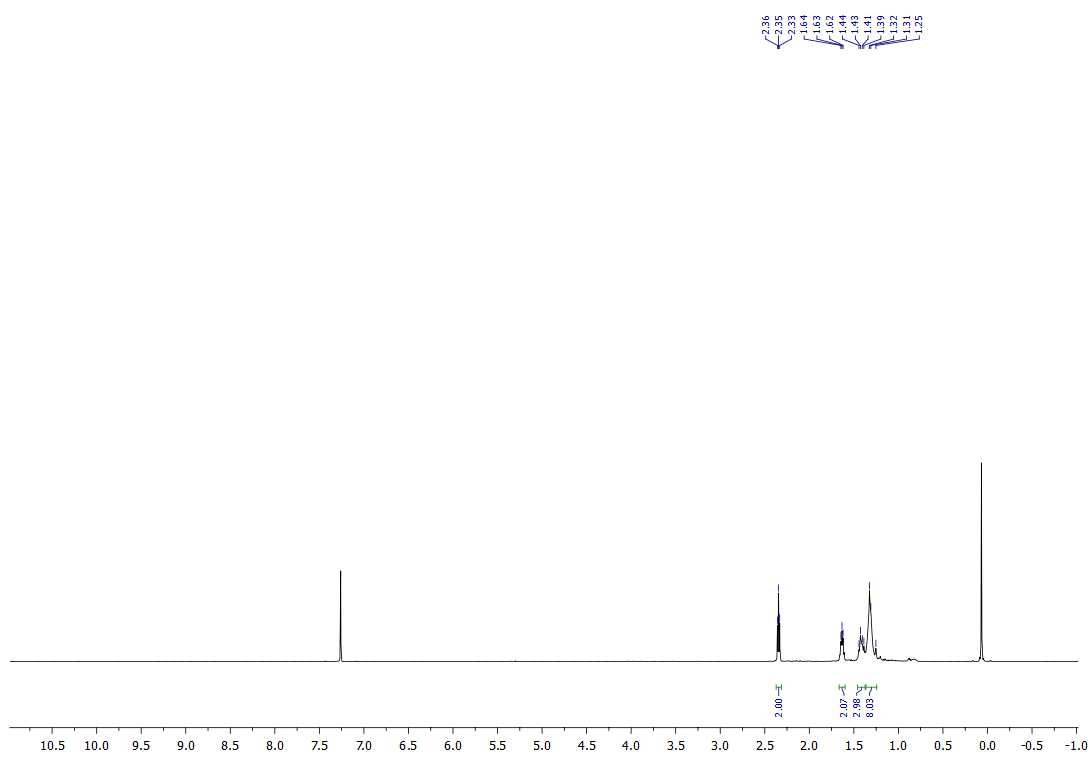

Supplement: Supplementary file 1 — This file contains Supplementary Figs. 1–3, Table 1 and Methods (related to chemical synthesis). [file 41586_2022_4787_MOESM1_ESM.docx]
