# Supplementary material for: ATGL is a biosynthetic enzyme for fatty acid esters of hydroxy fatty acids
Source: Nature. 2022 Jun 8;606(7916):968–75. doi: 10.1038/s41586-022-04787-x (PMC9242854; doi:10.1038/s41586-022-04787-x)
Supplement: Supplementary file 2 — Reporting Summary [file 41586_2022_4787_MOESM2_ESM.pdf]

Corresponding author(s): Barbara B. Kahn

Last updated by author(s): Feb 28, 2022

Reporting Summary

Nature Portfolio wishes to improve the reproducibility of the work that we publish. This form provides structure for consistency and transparency in reporting. For further information on Nature Portfolio policies, see our Editorial Policies and the Editorial Policy Checklist.

Statistics

For all statistical analyses, confirm that the following items are present in the figure legend, table legend, main text, or Methods section.

n/a	Confirmed	
☐	☒	The exact sample size (*n*) for each experimental group/condition, given as a discrete number and unit of measurement
☐	☒	A statement on whether measurements were taken from distinct samples or whether the same sample was measured repeatedly
☐	☒	The statistical test(s) used AND whether they are one- or two-sided<br>*Only common tests should be described solely by name; describe more complex techniques in the Methods section.*
☒	☐	A description of all covariates tested
☐	☒	A description of any assumptions or corrections, such as tests of normality and adjustment for multiple comparisons
☐	☒	A full description of the statistical parameters including central tendency (e.g. means) or other basic estimates (e.g. regression coefficient) AND variation (e.g. standard deviation) or associated estimates of uncertainty (e.g. confidence intervals)
☐	☒	For null hypothesis testing, the test statistic (e.g. *F*, *t*, *r*) with confidence intervals, effect sizes, degrees of freedom and *P* value noted<br>*Give P values as exact values whenever suitable.*
☒	☐	For Bayesian analysis, information on the choice of priors and Markov chain Monte Carlo settings
☒	☐	For hierarchical and complex designs, identification of the appropriate level for tests and full reporting of outcomes
☒	☐	Estimates of effect sizes (e.g. Cohen's *d*, Pearson's *r*), indicating how they were calculated

Our web collection on statistics for biologists contains articles on many of the points above.

Software and code

Policy information about availability of computer code

Data collection	Agilent 6470 Triple Quad Masshunter 10.0, Q-Exactive quadrupole-orbitrap, Li-cor Odyssey CLx
Data analysis	RawConverter 1.1.0.18,ProLuCID 1.4.2, DTASelect 2.1.4, LipidCreator 1.1.0, Skyline 20.2.0.286, Masshunter 10.0, Image Studio™Lite, GraphPad Prism 6.0 and 8.0

For manuscripts utilizing custom algorithms or software that are central to the research but not yet described in published literature, software must be made available to editors and reviewers. We strongly encourage code deposition in a community repository (e.g. GitHub). See the Nature Portfolio guidelines for submitting code & software for further information.

Data

Policy information about availability of data

All manuscripts must include a data availability statement. This statement should provide the following information, where applicable:

- Accession codes, unique identifiers, or web links for publicly available datasets
- A description of any restrictions on data availability
- For clinical datasets or third party data, please ensure that the statement adheres to our policy

We included the following Data availability statement under additional information section. The datasets and materials generated during the current study are available from the corresponding author (BBK) bkahn@bidmc.harvard.edu on reasonable request.

Field-specific reporting

Please select the one below that is the best fit for your research. If you are not sure, read the appropriate sections before making your selection.

☒ Life sciences ☐ Behavioural & social sciences ☐ Ecological, evolutionary & environmental sciences

For a reference copy of the document with all sections, see nature.com/documents/nr-reporting-summary-flat.pdf

Life sciences study design

All studies must disclose on these points even when the disclosure is negative.

Sample size	No sample size predetermination was performed. Sample size was chosen based on previous experience regarding the number of samples that would yield statistical significance. Citation: Yore et al. (2014)
Data exclusions	No data were excluded.
Replication	All experiments were repeated with similar results two- or three times. There were multiple replicates per experiment. The in-vivo biosynthesis study was performed on only one cohort of mice.
Randomization	We investigated changes in FAHFA synthesis or levels under well-controlled experimental conditions (i.e inhibition, loss or gain of function), therefore randomization is generally not applicable to these type of studies. We randomized the order in which we ran the samples on Mass Spec.
Blinding	Blinding was not applicable in our study. Blinding during collection was not needed because conditions were well controlled. Blinding during analysis was not feasible as the differences between samples under different conditions were visually apparent on Mass-spec runs. Blinding is also not necessary because the results are quantitative and did not require subjective judgment or interpretation. Blinding is not typically used in the field.

Reporting for specific materials, systems and methods

We require information from authors about some types of materials, experimental systems and methods used in many studies. Here, indicate whether each material, system or method listed is relevant to your study. If you are not sure if a list item applies to your research, read the appropriate section before selecting a response.

Materials & experimental systems

n/a	Involved in the study	
☐	☒	Antibodies
☐	☒	Eukaryotic cell lines
☒	☐	Palaeontology and archaeology
☐	☒	Animals and other organisms
☐	☒	Human research participants
☒	☐	Clinical data
☒	☐	Dual use research of concern

Methods

n/a	Involved in the study	
☒	☐	ChIP-seq
☒	☐	Flow cytometry
☒	☐	MRI-based neuroimaging

Antibodies

Antibodies used	ATGL (Cell Signaling # 2439, 30A4, 1:2000), GAPDH (Cell Signaling # 2118 , 14C10, 1:5000), DGAT1 and DGAT2 (gift from Robert V. Farese's Lab, PMID: 30936184)IR Dye 800CW anti-rabbit and anti-mouse secondary antibodies (LI-COR). This is included in the Methods western blotting section.
Validation	ATGL and GAPDH were commercial antibodies. DGAT1 and DGAT2 antibodies were generated and validated in a publication by Robert V. Farese's Lab, PMID : 30936184. These antibodies were only used for western blots with validation procedures described on the manufactures web sites or publication.<br>ATGL: https://www.cellsignal.com/products/primary-antibodies/atgl-30a4-rabbit-mab/2439?site-search-type=Products&N=4294956287&Ntt=atgl&fromPage=plp<br>The manufacturer's website lits 89 publications have used this antibody.<br>Additionally, we performed a confirmatory western blot with WT and AT-ATGL KO adipose tissue to validate specificity of the antibody.<br>GAPDH: https://www.cellsignal.com/products/primary-antibodies/gapdh-14c10-rabbit-mab/2118<br>The manufacturer's website lits 5360 publications have used this antibody. The web page also indicates," GAPDH (14C10) Rabbit mAb detects endogenous levels of total GAPDH protein."

Eukaryotic cell lines

Policy information about cell lines

Cell line source(s)	HEK 293T (ATCC), 3T3L1 (ATCC), HEPG2 (ATCC)
Authentication	All cell lines were purchased from commercial sources so we did not authenticate them further.
Mycoplasma contamination	All cell lines were tested and were negative for mycoplasma contamination.
Commonly misidentified lines (See ICLAC register)	We have not used commonly misidentified cell line in the study

Animals and other organisms

Policy information about studies involving animals; ARRIVE guidelines recommended for reporting animal research

Laboratory animals	AG4OX mice on FVB background, Control WT, AT-ATGL KO mice and control FL/FL mice on C57BL/6 background are described in methods. Male and female mice, age 8-12 weeks were used. Mice were housed at the Beth Israel Deaconess Medical Center at 23.3° C temperature and 40-60% humidity, on ventilated racks (25 ACH) under a 12h/12h light/dark cycle and fed on chow diet (Lab Diet, 5008). The housing conditions of the mice is included in the method mouse studies section.
Wild animals	No wild animals were used in the study.
Field-collected samples	No field collected samples were used in the study.
Ethics oversight	All experimental procedures were approved by the Institutional Animal Care and Use Committee of Beth Israel Deaconess Medical Center (BIDMC) and performed following its policies

Note that full information on the approval of the study protocol must also be provided in the manuscript.

Human research participants

Policy information about studies involving human research participants

Population characteristics	We receive de-identified samples from BNORC core, thus we do not have population charateristics
Recruitment	Adipose samples were obtained with informed consent from individuals undergoing surgery at Boston University Medical Center
Ethics oversight	All adipose tissue donors signed an informed consent form approved by the Boston Medical Center and Boston University Medical Campus Institutional Review Board. The use of de-identified human samples for this study was approved by the Institutional Review Board at Beth Israel Deaconess Medical Center.

Note that full information on the approval of the study protocol must also be provided in the manuscript.
